# Mid-infrared vision sensor based on suspended perovskite oxides down to nanometer scale

**DOI:** 10.1093/nsr/nwag499

**Published:** 2026-08-17

**Authors:** Haozhe Li, Haoran Mu, Wenzhi Yu, Yang Zhou, Kaiwen Gong, Yun Li, Jianxian He, Chen Chen, Jinkui Zhao, Daohua Zhang, Xiaoxue Song, Yuan Yang, Fang Wang, Guanyu Zhang, Shenghuang Lin

**Affiliations:** School of Integrated Circuits, Xi’an University of Technology, Xi’an 710048, China; Songshan Lake Materials Laboratory, Dongguan 523808, China; Songshan Lake Materials Laboratory, Dongguan 523808, China; School of Microelectronics Science and Technology, Sun Yat-sen University, Zhuhai 519082, China; Siscan Technology Co., Ltd. Suzhou 215100, China; Songshan Lake Materials Laboratory, Dongguan 523808, China; School of Integrated Circuits, Xi’an University of Technology, Xi’an 710048, China; Songshan Lake Materials Laboratory, Dongguan 523808, China; State Key Laboratory of Advanced Technology for Materials Synthesis and Processing, Wuhan University of Technology, Wuhan 430070, China; Songshan Lake Materials Laboratory, Dongguan 523808, China; School of Physical Sciences, Great Bay University, Dongguan 523000, China; Songshan Lake Materials Laboratory, Dongguan 523808, China; School of Physical Sciences, Great Bay University, Dongguan 523000, China; Institute of Physics, Chinese Academy of Sciences, Beijing 100190, China; School of Integrated Circuits, Xi’an University of Technology, Xi’an 710048, China; Shenzhen Pinghu Laboratory, National Wide Bandgap Semiconductor Technology Innovation Center, Shenzhen 518111, China; Jiangsu Institute of Advanced Semiconductors LTD, Suzhou 215123, China; School of Integrated Circuits, Xi’an University of Technology, Xi’an 710048, China; Shanghai Institute of Technical Physics, Chinese Academy of Sciences, Shanghai 200083, China; Songshan Lake Materials Laboratory, Dongguan 523808, China; Institute of Physics, Chinese Academy of Sciences, Beijing 100190, China; Songshan Lake Materials Laboratory, Dongguan 523808, China

**Keywords:** mid-infrared detector array, photothermoelectric effect, suspended SrTiO_3_ membrane, programmable vision sensor, in-sensor computing

## Abstract

Intelligent mid-infrared (MIR) imaging integrates in-sensor preprocessing of thermal information, thereby reducing data redundancy and improving target-recognition robustness under low-visibility conditions. However, unlike in the visible regime, photoconductive programmability in the MIR typically relies on narrow-bandgap materials, which inherently limit stable and reconfigurable operation. Here, we report a programmable photothermoelectric detector based on a suspended nanometer-thick SrTiO_3_ membrane. Dimensional scaling of the thermoelectric channel enhances sensitivity and enables a response time of ∼10 μs, over 10^4^ times faster than bulk SrTiO_3_. Through dual-gate electrostatic modulation of the Seebeck profile, the device exhibits bidirectionally tunable photothermoelectric responsivity up to 50 V W^−1^ and supports 40 experimentally observed programmable response states. By leveraging a 3 × 3 programmable device array with device-to-device consistent gate-programmable photoresponse and reproducible polarity switching, a recognition accuracy of 83% is achieved under visually degraded conditions. Furthermore, the programmable array enables attention-guided thermal image weighting by enhancing target thermal signatures while suppressing background interference. These results demonstrate the dimensional scaling of perovskite oxides as an effective route toward adaptive, low-power machine vision and neuromorphic infrared electronics.

## Teaser

Dimensional scaling of suspended SrTiO3 membranes enables mid-infrared photothermoelectric sensing with high responsivity (>50 V W^−1^), fast response (∼10 μs), and gate programmability for convolutional image preprocessing and adaptive thermal-vision.

## INTRODUCTION

Intelligent mid-infrared (MIR) imaging enables target recognition by sensing thermal radiation rather than reflected light, making it essential for applications such as autonomous navigation, night vision, and environmental monitoring. Programmable infrared photodetectors capable of reconfigurable operation are essential for next-generation vision and intelligent sensing, enabling in-sensor computing, multifunctional imaging, and self-adaptive optoelectronics [1–6]. While progress has been made in the visible and near-infrared domains, extending these capabilities into the mid-wave (MWIR, 3–5 μm) and long-wave (LWIR, 8–12 μm) infrared bands remains challenging, particularly given the simultaneous demands for high sensitivity, fast response and device miniaturization [7,8]. Photon-type infrared detectors, typically based on narrow-bandgap semiconductors such as HgCdTe [9,10] and InGaAs [11], offer high responsivity and broad spectral coverage. However, maintaining high detectivity often necessitates cryogenic cooling to suppress thermally generated dark current, especially in the LWIR band, which increases power consumption and system complexity, thereby limiting scalability and reconfigurability [12,13]. Moreover, their spectral response, intrinsically fixed by material composition, constrains adaptability across the infrared spectrum [14,15]. Emerging self-powered photovoltaic-type infrared detectors based on the bulk photovoltaic effect, polarization fields, or depolarization-field-driven mechanisms have also been explored in systems such as WS_2_ nanotubes, twisted graphene, rhombohedral MoS_2_, and Janus MoSSe [16–20]. However, because these mechanisms still rely on photon absorption and the separation of photogenerated carriers, their extension to longer infrared wavelengths is generally constrained by inefficient low-energy photon absorption or narrow-bandgap-induced dark current and noise, limiting stable, low-noise, and reconfigurable MIR operation.

Thermal detectors represent another important class of infrared sensors, typically relying on chalcogenide alloys such as Bi_2_Te_3_ [21] and PbTe [22], or oxide thermoelectrics, including SrTiO_3_, BaTiO_3_, and CaMnO_3_ [23]. These materials can operate at room temperature and inherently support broadband infrared detection, offering advantages in power efficiency and structural simplicity. However, their response speed is generally limited by phonon-mediated heat transport, resulting in slow temporal dynamics and restricted modulation capability [24]. Consequently, the concept of reconfigurable or programmable responsivity has rarely been explored in thermal-type infrared detectors. SrTiO_3_, as a representative perovskite oxide, combines a large Seebeck coefficient (up to −10^3^ μV K^−1^) with excellent chemical robustness, thermal stability, and environmental compatibility [25]. Photothermoelectric (PTE) detectors based on bulk SrTiO_3_ have demonstrated photovoltages exceeding 10 mV and photocurrents reaching the microampere (μA) level under infrared illumination, corresponding to responsivities of up to 1.2 V W^−1^ at 10.7 μm [26,27]. Moreover, SrTiO_3_ maintains linear performance over a wide power range, up to 1235 W cm^−2^, which is critical for high-intensity infrared sensing. Nevertheless, the use of bulk SrTiO_3_ inherently limits response speed (∼1.5 s) and lacks gate tunability, restricting its reconfigurable and programmable functionality [26].

Here, we report a programmable infrared PTE detector based on a suspended nanometer-thick SrTiO_3_ membrane. The device achieves broadband self-powered photoresponse from the visible to the long-wave infrared region with a response time of ∼10 μs. With dual local gates enabling independent electrostatic doping, the responsivity can be further enhanced to 50 V W⁻¹ and 37 experimentally observed programmable response states with reversible polarity control are achieved. Furthermore, a 3 × 3 programmable device array exhibits device-to-device consistent gate-programmable photoresponse. Benefiting from the wavelength-independent responsivity and excellent linearity across input power levels, the detector array can be programmed to simultaneously sense and process images with reconfigurable kernels. Even under 10% ambient brightness, the SrTiO_3_ PTE detector maintains 83% recognition accuracy, over twice that of a visible-domain programmable image sensor. This work establishes programmable photothermoelectricity in suspended nanometer-thick perovskite oxides as a foundation for reconfigurable infrared imaging and in-sensor computing across infrared spectral ranges.

## RESULTS AND DISCUSSION

### Device structure and intrinsic material properties

**Figure 1.**
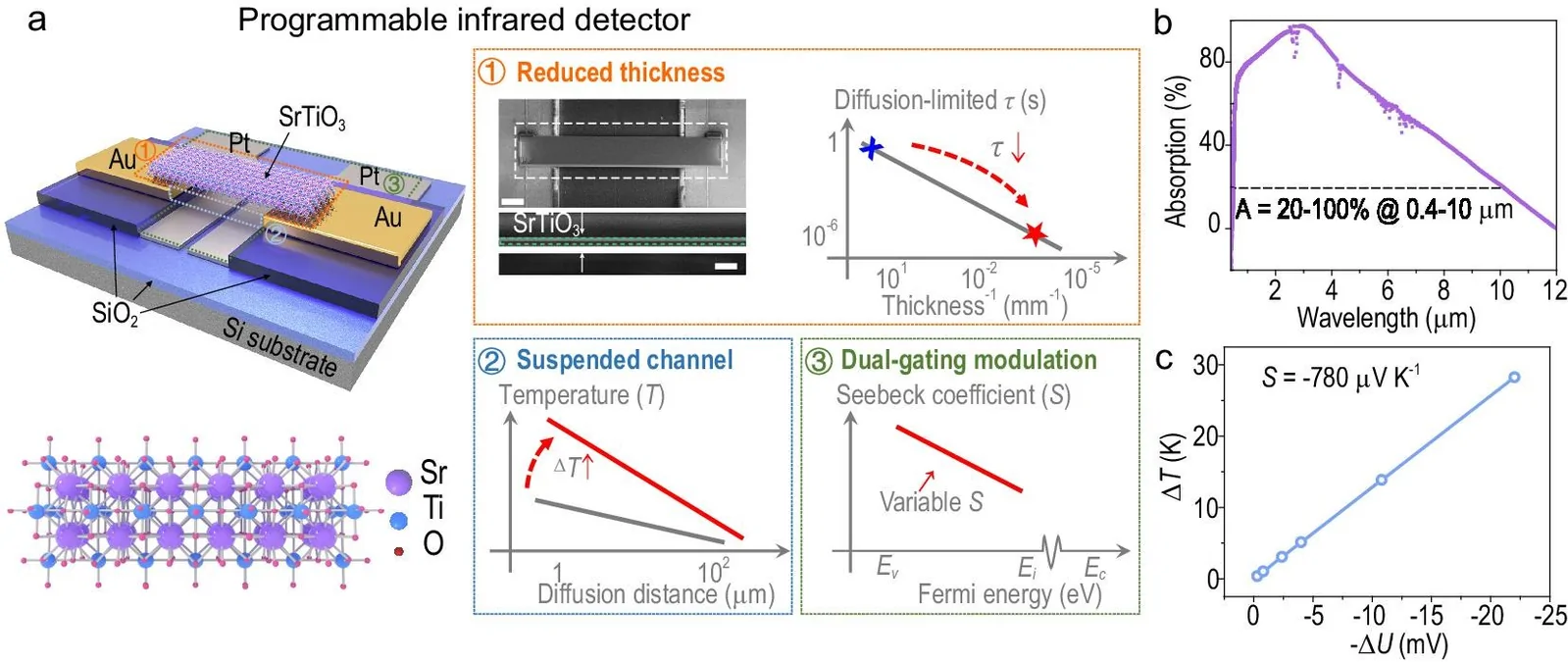
Device concept and material properties of the suspended nanometer-thick SrTiO_3_. (a) Schematic illustration of the suspended SrTiO_3_ PTE detector. The crystal structure of SrTiO₃ is shown below. The three design principles are illustrated on the right. (1) The thin SrTiO_3_ layer (SEM top and cross-sectional views) substantially reduces the thermal diffusion time constant, as evidenced by the strong thickness dependence extracted from both literature data (blue) and this work (red). (2) The suspended channel preserves the lateral temperature gradient. (3) Dual-gate modulation enables local tuning of the Seebeck coefficient. (b) Optical absorption spectrum of SrTiO_3_. (c) Measured thermal voltage across the SrTiO_3_ channel as a function of the Seebeck coefficient at room temperature, used to derive the temperature gradient.

Figure 1a illustrates the design concept of the programmable MIR PTE detector. The fabrication process is depicted in Fig. S1. The suspended SrTiO_3_ thin film serves as the thermoelectric channel, as highlighted in an orange dashed box. Top-view and cross-sectional scanning electron microscope (SEM) images reveal a well-defined rectangular geometry with a thickness of ∼230 nm. To directly verify that the SrTiO_3_ membrane is suspended, more top-view, tilted-view (52° stage tilt) and cross-sectional SEM characterization is shown in Fig. S2. These images confirm that the SrTiO_3_ membrane bridges between the two electrodes without bending and collapse and is separated from the underlying substrate by the air gap, confirming the suspended geometry and indicating sufficient mechanical robustness for the device measurements. The membrane thickness is more than two orders of magnitude smaller than that of previously reported SrTiO_3_-based PTE devices [14]. Such thickness scaling substantially reduces the areal heat capacity and active thermal mass, contributing to a shortened thermal relaxation time [26–29]. In addition, the suspended membrane architecture suppresses heat leakage to the substrate and helps preserve lateral temperature gradients, further enhancing the PTE response (see the mechanism section). Crucially, the nanometer-thick membrane also facilitates electrostatic modulation of the Seebeck profile, which is essential for reconfigurable PTE detection [30,31]. To enable programmable operation, dual bottom gates are integrated beneath the suspended channel to locally modulate the carrier density and spatial distribution of the Seebeck coefficient.

The crystallinity and orientation of the SrTiO_3_ single crystals prior to thinning were characterized by X-ray diffraction. A distinct (200) reflection at 2θ = 46.3° confirms the high crystalline quality and indicating that the fabricated thin film is oriented along the (100) plane of the cubic perovskite structure (space group Pm-3m). Relative to undoped SrTiO_3_, the (200) peak shows a shift of 0.1°–0.2° toward lower angles, consistent with lattice expansion induced by 0.5% Nb doping [32] (Fig. S3). Infrared optical absorption measurements further reveal strong sub-band-gap absorption exceeding 20% and extending up to 10 μm (Fig. 1b). The Seebeck coefficient was assessed by applying a controlled temperature gradient across the SrTiO_3_ single crystal and recording the induced voltage. A linear dependence of voltage (Δ*U*) on temperature difference (Δ*T*) was observed, yielding a Seebeck coefficient of ∼−780 μV K^−1^ (Fig. 1c). We note that direct measurement of the Seebeck coefficient of the suspended nanometer-thick membrane is technically challenging because of its small lateral size and ultrathin geometry. Nevertheless, the suspended membrane was cut and released from the same single crystal, and therefore it is expected to retain a comparable thermoelectric response to that of the parent crystal.

### Photoelectronic behavior without gate modulation

**Figure 2.**
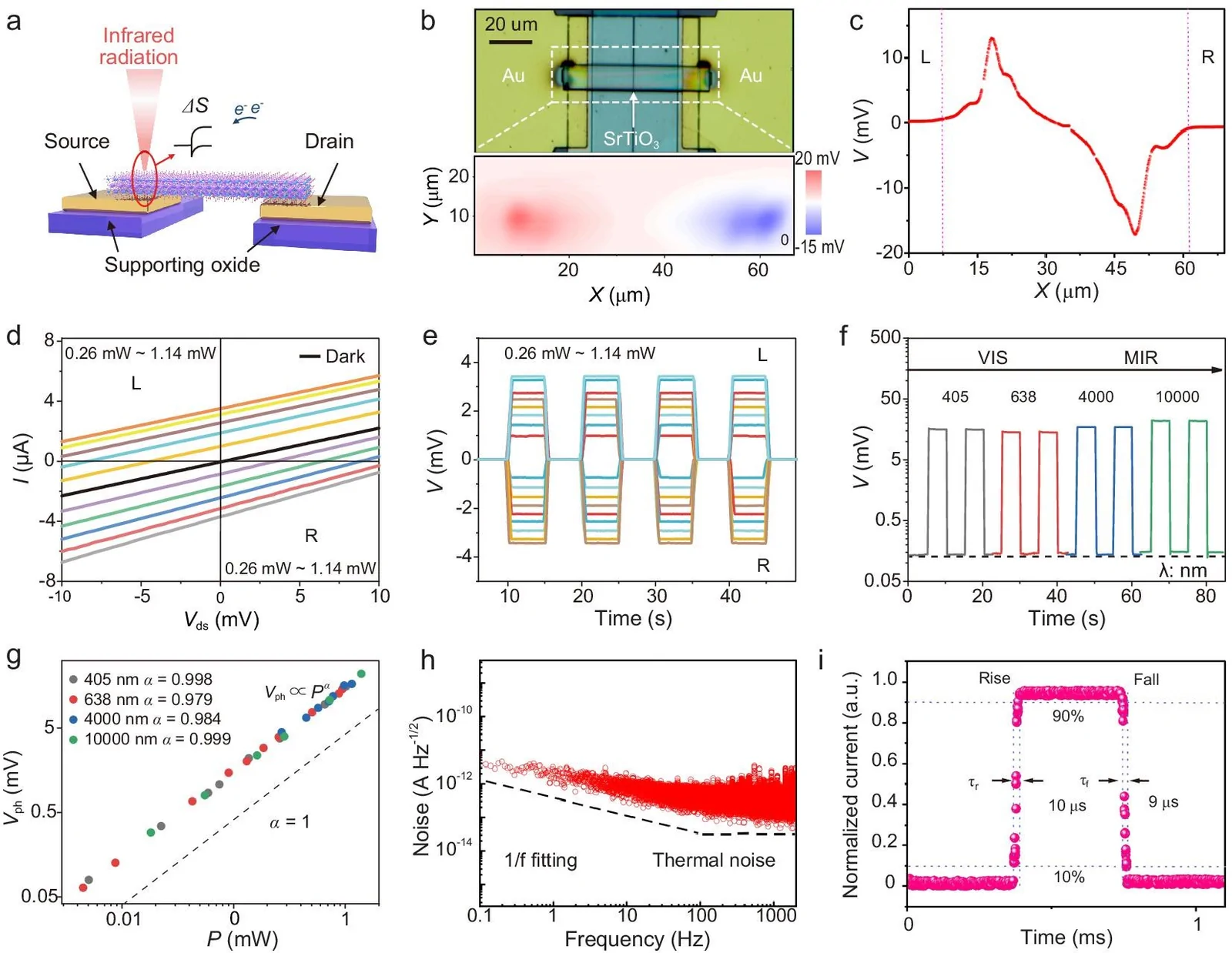
Ungated interface-dominated photoresponse in the suspended SrTiO_3_ PTE detector. (a) Schematic illustration of the device architecture, in which the incident infrared radiation is focused near the metal–SrTiO_3_ junction to maximize the PTE current. (b) Optical micrograph of the SrTiO_3_ device and corresponding spatially resolved photocurrent mapping result. The white dashed box marks the region scanned for photocurrent mapping and the laser wavelength is 4 μm, measured at zero source–drain bias. (c) Linear photocurrent scan. (d) *I*–*V* curves under left- and right-end illumination at various optical powers. (e) Temporal photovoltage response under left- and right-side illumination at different optical powers, measured at zero source–drain bias. (f) Photovoltage response across the visible, near-infrared, and MIR spectral bands. (g) Photovoltage as a function of optical power with power-law fitting. (h) Noise current data measured using a phase-locked amplifier. (i) Time-resolved photoresponse, measured at zero source–drain bias.

The PTE response of the suspended SrTiO_3_ thin film was first characterized under zero gate bias. A 4 μm infrared laser was focused near the metal–SrTiO_3_ junction, where interfacial band bending enhances local heating and maximizes the PTE response (Fig. 2a). The corresponding optical micrograph is shown in the top panel of Fig. 2b. Spatial photocurrent mapping of the region outlined by the dashed box reveals PTE voltages of comparable magnitude but opposite polarity at the left and right metal contacts. A photovoltage profile extracted by scanning the focused laser spot along the channel from source to drain (Fig. 2c) shows pronounced extrema at the two metal interfaces, while the response vanishes near the channel midpoint. This spatial signature is consistent with PTE generation driven by lateral temperature gradients under localized excitation.

Figure 2d presents the *I*–*V*_ds_ characteristics of the suspended SrTiO_3_ thin film detector. In the dark, the device displays a linear current–voltage relation with negligible offset, confirming ohmic contacts between the channel and electrodes. Under focused laser excitation near the left (L) or right (R) electrode, the *I*–*V* curves exhibit a horizontal shift without a change in slope, consistent with PTE generation. The invariance of the slope under illumination excludes contributions from photoconductive and bolometric effects. The shift amplitude increases monotonically with optical power, producing a zero-bias photocurrent of ∼4 μA under 1.14 mW excitation, corresponding to a photovoltage exceeding 15 mV. Time-resolved measurements under periodic illumination further confirm self-powered operation without external bias (Fig. 2e). Opposite polarities are observed when the excitation is applied near the L or R electrode, in agreement with the *I*–*V* characteristics. The response remains stable and reproducible over repeated cycles, demonstrating reliable device performance. We note that no sharp transient voltage or current spikes are observed at the light on/off transitions in the time-dependent photoresponse measurements, suggesting that strain-gradient-induced flexoelectric, photoflexoelectric, or pyroelectric-like effects do not play a dominant role in the present devices.

Broadband responsivity under self-powered operation is a key characteristic of PTE detectors. The suspended SrTiO_3_ thin-film device exhibits stable photovoltage responses under pulsed illumination at representative wavelengths of 405 nm, 638 nm, and 10 μm (Fig. S4). At a constant incident power of ∼1 mW, the output photovoltage remains nearly a constant at ∼15 mV across the visible to the long-wave infrared wavelengths (Fig. 2f). Owing to the negligible dark voltage, the corresponding light on/off ratio exceeds 250 throughout the spectral window. The dependence of photovoltage on optical power follows a power-law relation *V*_ph_ ∝ *P*^α^, where *P* is the incident optical power and α is the ideality factor. Fitted values of α are consistently above 0.979 for all measured wavelengths, confirming nearly ideal linearity over more than two orders of magnitude in optical power (Fig. 2g). Such linearity is critical for programmable operation, as it enables predictable and scalable response states.

The sensitivity of the suspended SrTiO_3_ PTE detector was further evaluated. The noise spectral density exhibits 1/*f*-dominated behavior at low frequencies, which gradually approaches the thermal noise limit at higher frequencies (Fig. 2h). The responsivity, defined as *R* = *V*_ph_/*P*, maintaining uniformly high at >13 V W^−1 ^ across the spectral range from 0.4 to 10 μm (Fig. S4). No saturation of the temperature difference is observed even at high power densities up to 1.54 × 10^6^ mW cm^−2^, indicating a wide linear dynamic range. Based on the measured noise and responsivity, the noise-equivalent power (NEP) is given by


(1) \begin{eqnarray*}
{\mathrm{NEP}} = \frac{{\sqrt {{{\left\langle {{i}_{\mathrm{n}}} \right\rangle }}^2} }}{R},
\end{eqnarray*}

where ${\langle {{i}_{\mathrm{n}}} \rangle }^2$ is the current noise spectral density. The NEP remains nearly constant over all different powers and wavelengths, with a minimum of ∼230 pW Hz^−1/2^ (Fig. S5). Considering the calibrated beam diameters of ∼15.8, 14.2, 15.1, and 29.4 μm for 405, 638, 1550 and 4000 nm, respectively (Fig. S6), specific detectivity is estimated to be ∼3 × 10^8^ Jones, according to ${D}^* = \frac{{\sqrt A }}{{{\mathrm{NEP}}}}$, where *A* is the effective photosensitive area and NEP is the noise-equivalent power. These performance metrics surpass those of bulk SrTiO_3_ and most previously reported PTE detectors employing diverse channel materials and architectures (Table S1).

The transient photoresponse of the suspended SrTiO_3_ thin-film detector was characterized under square-pulse modulation, as shown in Fig. 2i. By using an optimized preamplified readout, a single ON/OFF cycle recorded by an oscilloscope gives a rise time (*τ*_rise_) of ∼10 μs and a fall time (*τ*_fall_) of ∼9 μs. These values are close to the bandwidth limit of the measurement setup, suggesting that the intrinsic device response could be even faster. The measured speed is more than four orders of magnitude higher than that of bulk SrTiO_3_ PTE detectors, and comparable to state-of-the-art layered material systems such as MoTe_2_ and black phosphorus for infrared detection (Table S1). This improvement arises from channel thinning, which reduces thermal mass and enables rapid thermal equilibration.

### Dual-gate programmable photoelectronic response

**Figure 3.**
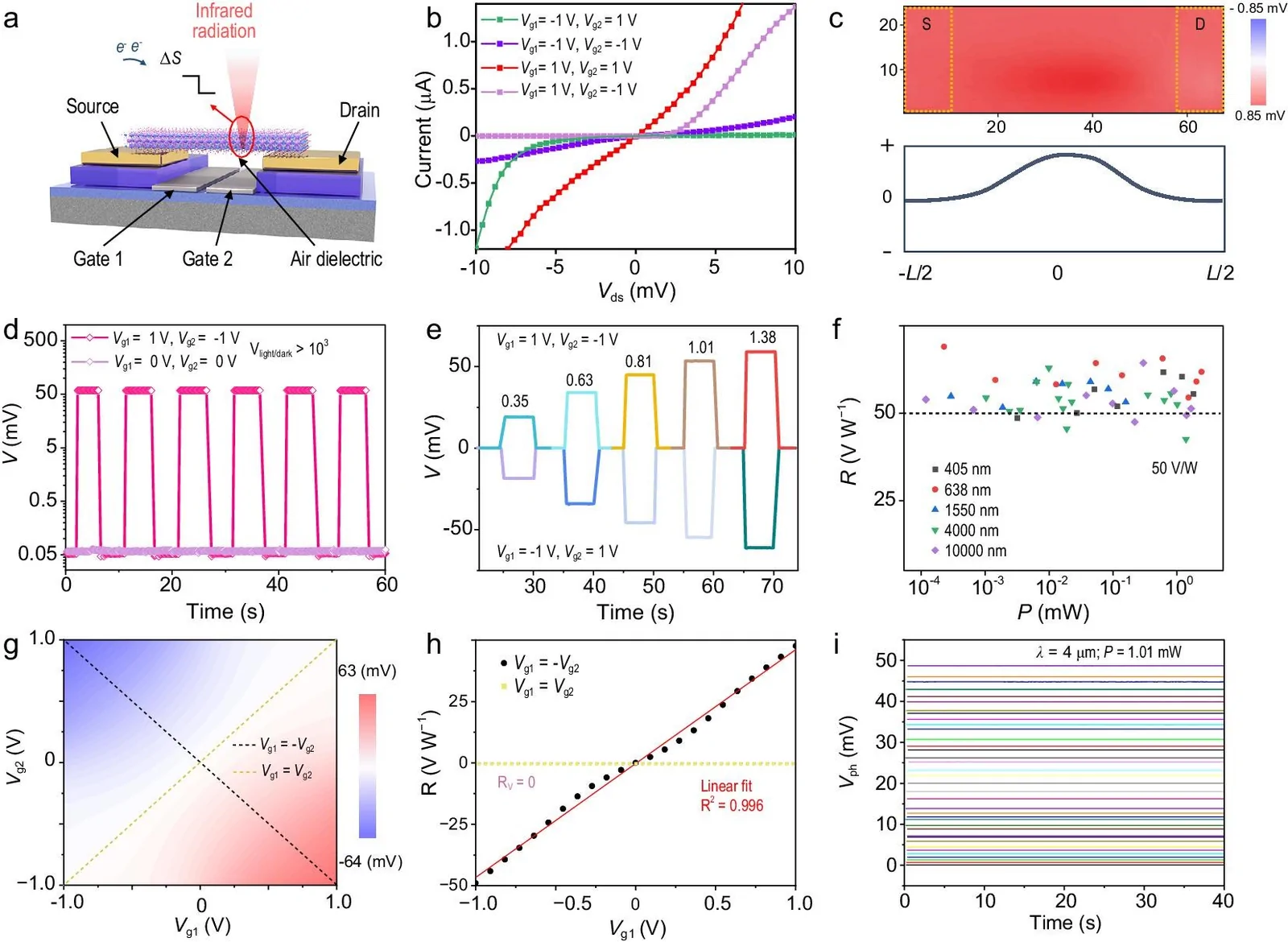
Dual-gate-programmed center-channel photoresponse in the suspended SrTiO_3_ PTE detector. (a) Schematic of the dual-gated SrTiO_3_ PTE device, with infrared radiation focused at the midpoint of the channel where the gate-induced Seebeck contrast (Δ*S*) maximizes the PTE response. (b) Dual-gate modulation of the dark *I*–*V* characteristics of suspended SrTiO_3_. Symmetric gating yields linear ohmic behavior with enhanced conductance under positive bias, whereas antisymmetric gating produces rectifying responses with opposite polarities, indicative of lateral junction formation between the two gated regions. (c) Spatial photovoltage map and line profile under antisymmetric bias *V*_g1_ = +1 V and *V*_g2_ = −1 V, showing enhanced center-channel response, measured at zero source–drain bias. (d) Time-resolved photovoltage at the channel center with and without antisymmetric gate modulation, measured at zero source–drain bias. (e) Time-resolved photovoltage at the channel center under different incident optical powers, measured at zero source–drain bias. (f) Responsivity as a function of incident optical power for wavelengths ranging from 405 nm to 10 μm, demonstrating a broadband and power-independent PTE response. (g) Two-dimensional photovoltage map as a function of *V*_g1_ and *V*_g2_ under 4 μm illumination, with symmetric and antisymmetric gate trajectories indicated. (h) Responsivity extracted along representative gate-bias trajectories. (i) Experimentally measured discrete photovoltage states under 4 μm illumination with a fixed illumination power, measured at zero source–drain bias.

As shown in Fig. 3a, the air gap serves as the dielectric medium, and independent electrostatic modulation is achieved through a dual split-gate configuration. Two individual bottom gates, Gate 1 and Gate 2, are positioned beneath the suspended SrTiO_3_ channel to independently tune the local carrier density and Fermi level in the two adjacent regions. In this measurement configuration, the infrared beam is focused at the center of the suspended channel rather than near the metal/SrTiO_3_ interface, so that the photoresponse is governed primarily by the gate-induced Seebeck contrast between the two gated regions. Figure 3b shows the dark *I*–*V* characteristics of the suspended SrTiO_3_ device under different dual-gate configurations. With symmetric gating (*V*_g1_ = *V*_g2_), the device exhibits nearly linear *I–V* relations, consistent with ohmic contacts. The conductance is markedly higher under positive bias (+1 V, +1 V) than under negative bias (−1 V, −1 V), reflecting the *n*-type nature of SrTiO_3_, where positive gate voltages shift the Fermi level toward the conduction band and increase carrier density. In contrast, antisymmetric gating (*V*_g1_ = +1 V, *V*_g2_ = −1 V and *V*_g1_ = −1 V, *V*_g2_ = +1 V) produces rectifying *I–V* curves with opposite polarities. This behavior indicates that the dual local gates generate laterally asymmetric carrier-density profiles in the suspended channel.

The photovoltage mapping result was performed under antisymmetric gating with *V*_g1_ = +1 V and *V*_g2_ = −1 V is shown in Fig. 3c. Unlike the ungated device, where the strongest response appears near the metal/SrTiO_3_ contact regions, the dual-gated device exhibits a pronounced photovoltage maximum at the center of the suspended channel. The corresponding line profile further confirms that the photoresponse is enhanced around the boundary between the two gated regions.

After confirming the channel-center photoresponse in the spatial map, we compared the time-resolved photovoltage under antisymmetric gating and zero-gate conditions. As shown in Fig. 3d, under zero gate bias, the device shows negligible signal, consistent with the spatial distribution of photovoltage in Fig. 2c. In contrast, applying antisymmetric gate voltages (*V*_g1_ = +1 V, *V*_g2_ = −1 V) produces a strong periodic response with a photovoltage exceeding 50 mV and an ON/OFF ratio >10^3^. This comparison demonstrates the significant enhancement of center-channel photoresponse by dual-gate modulation. Figure 3e shows stable and repeatable photovoltage switching as the incident infrared power is varied. The photovoltage amplitude increases monotonically increasing optical power, reaching ∼65 mV at 1.38 mW, while the polarity reverses when the gate voltage are interchanged (*V*_g1_ = −1 V, *V*_g2_ = +1 V). Broadband programmable response was evaluated, in which stable switching is observed from 405 nm to 10 μm (Fig. S7), confirming wavelength-independent behavior. Across all measured wavelengths, the *V*_ph_–*P* characteristics under antisymmetric gate voltages (*V*_g1_ = +1 V, *V*_g2_ = −1 V), extracted in Fig. S8, show a linear photovoltage response to incident laser power from 0.1 μW to 1 mW. The linear dynamic range is calculated to over 80 dB.

Based on the measured photovoltage and calibrated incident optical power, the voltage responsivity is remains stable at ∼50 V W^−1^ across the visible to long-wave infrared range under the +1/−1 V gate configuration (Fig. 3f). The NEP exhibits minimal variation across different incident power levels and wavelengths, with a minimum value of ∼18 pW Hz^−1/2^. The corresponding specific detectivity, D*, reaches ∼3 × 10^9^ Jones, as shown in Fig. S9.

The full dual-gate dependence of the center-channel photoresponse was further summarized by a two-dimensional *V*_g1_–*V*_g2_ photovoltage map under fixed 4 μm infrared illumination. As shown in Fig. 3g, the photovoltage is strongly suppressed along the symmetric-gating direction (*V*_g1_ = *V*_g2_, black line), whereas it is maximized and sign-reversible along the antisymmetric-gating direction (*V*_g1_ = −*V*_g2_, red line). This result indicates that the center-channel photoresponse is mainly determined by the relative electrostatic difference between the two gated regions. The corresponding raw gate-sweep curves are shown in Fig. S10, where *V*_g1_ is swept from −1 to +1 V, while *V*_g2_ is stepped from −1 to +1 V.

To quantitatively evaluate the programmability, the voltage responsivity was measured along the two-representative gate-bias trajectories in Fig. 3h. Along the antisymmetric-gating trajectory (*V*_g1_ = −V_g2_), the voltage responsivity varies nearly linearly from negative to positive values within ∣*V*_g_∣ < 1 V, consistent with the electrostatically modulated Seebeck profile. In contrast, along the symmetric-gating trajectory (*V*_g1_ = *V*_g2_), the responsivity remains close to zero. This continuous tunability enables the definition of more than 3300 distinct photoresponse states (Note S3).

To further demonstrate experimentally accessible multilevel programmability, discrete gate-bias configurations were selected along the programmable response range and the corresponding photovoltage states were recorded under fixed 4 μm illumination with an incident power of 1.01 mW. As shown in Fig. 3i, 40 stable photovoltage states are experimentally obtained, spanning from nearly zero to ∼50 mV. Each state remains nearly constant during the measurement, indicating stable gate-defined output levels rather than transient fluctuations. The voltage separation between adjacent states is much larger than the fluctuation within each state, suggesting that the experimentally demonstrated 40 states do not represent the upper limit and that additional programmable states could be accommodated by using finer gate-voltage steps.

### PTE evidence and programmable response mechanism

We first verify that the observed infrared photoresponse is dominated by the PTE effect rather than by photovoltaic, photoconductive, or bolometric mechanisms. Under infrared illumination, the *I*–*V* curves remain nearly linear with almost unchanged slopes but exhibit a clear voltage offset, providing primary evidence that the photoresponse is not dominated by bolometric or photoconductive effects, which would mainly alter the channel conductance. In addition, the device exhibits broadband response from the visible to the midwave infrared regions, where the photon energy is far below the bandgap of SrTiO_3_, making direct interband photovoltaic generation unlikely to be the dominant mechanism. The comparable responsivity at 0.4, 0.6, 4.0, and 10.0 μm further supports a thermally mediated photoconversion process rather than a photon-type response governed by band-to-band absorption. Spatially resolved photovoltage mapping reveals opposite response polarities near the two electrodes and a suppressed signal near the channel center in the ungated device, consistent with local-temperature-gradient-driven thermoelectric voltage generation. These observations support that the infrared photoresponse is dominated by the PTE effect.

The fast and enhanced photoresponse can be understood from the reduced thermal capacitance and modified heat-dissipation pathway of the nanometer-thick suspended SrTiO_3_ membrane. As discussed in Note S1, the areal heat capacity scales with membrane thickness, reducing the SrTiO_3_ channel to the nanometer scale markedly decreases the thermal mass that must be heated and cooled during optical excitation, thereby increasing the light-induced temperature rise and accelerating thermal relaxation. Different from conventional Si-based or 2D materials-based photovoltaic photodetectors, whose response speed can often be limited by the electrical *RC* time constant, the temporal response of our PTE device is much longer than the *RC* time constant and is instead governed by thermal relaxation. As shown in Fig. S11, the measured capacitance is on the order of pF. Considering a device resistance of ∼5000 Ω, extracted from the measured *I*–*V* curve, the corresponding electrical *RC* time constant is estimated to be on the order of 10 ns. This value is far shorter than the measured optical response time in the microsecond range, confirming that the response speed is not limited by electrical *RC* charging.

In addition, as shown in Fig. S14, we measured the response times of all devices in the array and summarized the corresponding statistics. The results show that thinner membranes generally exhibit faster photoresponse. Because all devices were measured using the same readout chain, this thickness-dependent trend indicates that the measured response time is not governed by a fixed instrumental or readout-bandwidth limit, but varies with the membrane thickness. This provides experimental support that reducing the membrane thickness and thermal capacitance contributes to faster thermal relaxation. We also clarified that the effective thermal time constant cannot be described by a simple thickness-only scaling model, as it also depends on thermal boundary resistance, heat dissipation through the electrodes and supporting regions, and possible radiative or convective losses from the suspended structure.

**Figure 4.**
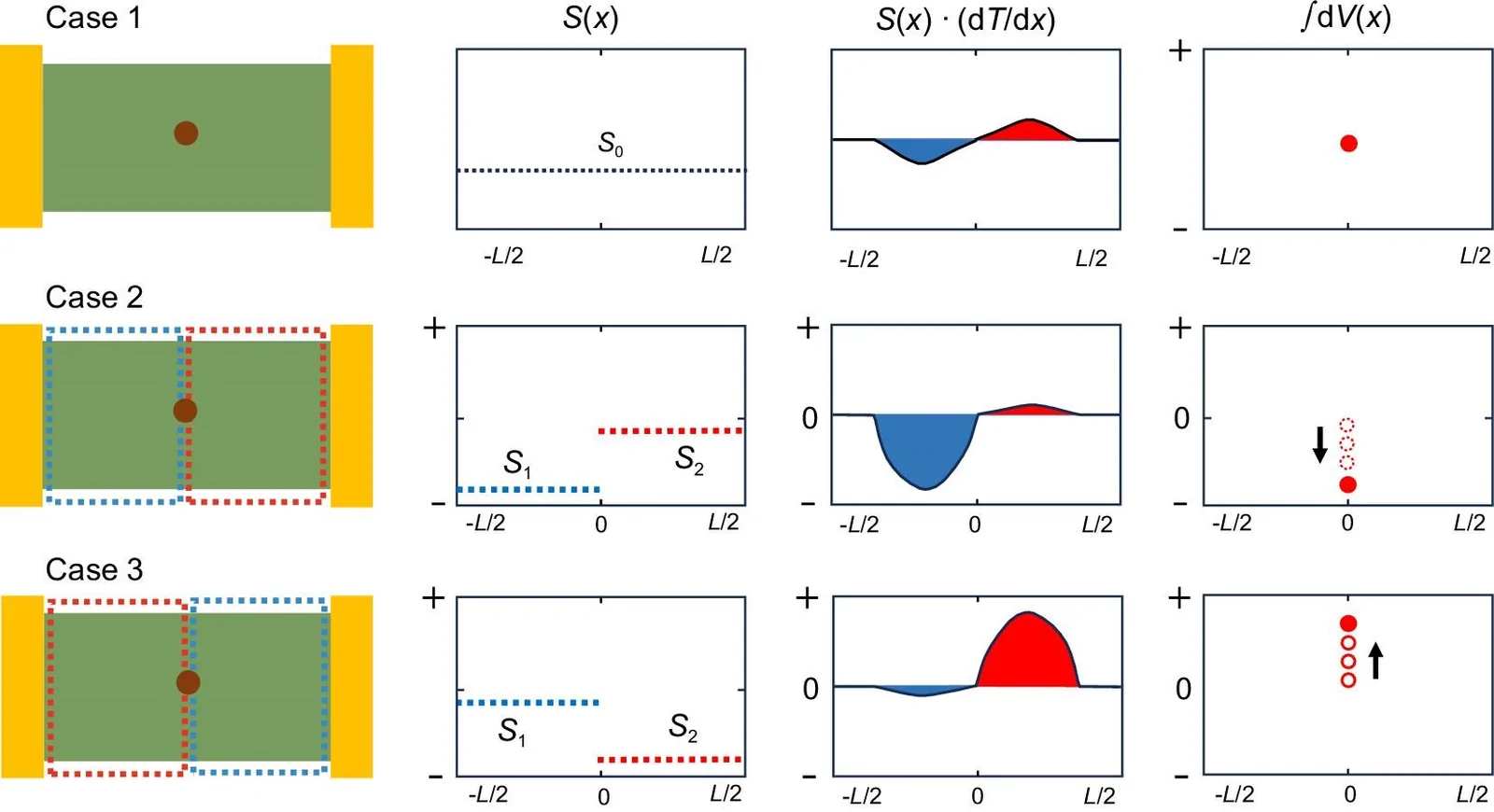
Mechanism of programmable PTE response in suspended SrTiO_3_. From left to right: device geometry under laser illumination, Fermi-level distribution modulated by dual gates, local photovoltage contribution ${\rm d}V( x ) = S( x )\,\frac{{dT}}{{dx}}\,{\rm d}x$, and the integrated net photovoltage. Top row: without gating, the uniform Fermi level yields a constant Seebeck coefficient and antisymmetric ${\rm d}V( x )$ contributions that cancel, giving negligible net output. Middle row: under antisymmetric gating (+1 V, –1 V), the asymmetric Seebeck profile produces unbalanced ${\rm d}V( x )$ contributions and a net negative photovoltage. Bottom row: reversing the gate polarity (–1 V, +1 V) inverts the Seebeck profile and results in a positive photovoltage. The multiple discrete dots in the last column indicate the experimentally accessible tunable states, demonstrating continuous programmability beyond simple polarity reversal.

The programmable response originates from dual-gate control of the local Seebeck profile in the suspended SrTiO_3_ membrane. We correlate the spatial photocurrent mapping results (Fig. S12) with the schematic analysis in Fig. 4. Without gating, the suspended SrTiO_3_ channel exhibits a nearly uniform Fermi level, corresponding to a spatially constant Seebeck coefficient. Under local optical excitation, the lateral temperature gradient generates positive and negative contributions of the form [33–35]


(2) \begin{eqnarray*}
{V}_{ph} \propto \mathop \int \nolimits_{ - L/2}^{L/2} S(x) \cdot \left( {\frac{{dT}}{{dx}}} \right)dx,
\end{eqnarray*}

which are antisymmetric and cancel upon integration, yielding negligible net photovoltage (Fig. 4, top row). This behavior is consistent with the photocurrent mapping results, where the signal vanishes at the channel center.

When antisymmetric gate voltages are applied, the Fermi level shifts in opposite directions across the two halves of the channel, establishing an asymmetric Seebeck profile (Fig. 4, middle and bottom rows). In these cases, the contributions from the left (−*L*/2, 0) and right (0, *L*/2) halves no longer cancel but instead add up to produce a finite photovoltage. The effect is maximized when the laser is focused at the channel midpoint between the two split gates, where the Seebeck contrast is strongest. By varying antisymmetric gate voltages (*V*_g1_ = −*V*_g2_), the photovoltage magnitude can be continuously tuned.

The gate-programmed PTE responsivity follows ${R}_V \approx {K}_1( {{V}_{g1} - {V}_{g2}} ) + {K}_3( {V_{g1}^3 - V_{g2}^3} )$, linking the output response directly to the Seebeck contrast between the two gated channel regions. For small gate-voltage excursions, the Seebeck–gate dependence can be linearized, yielding ${R}_V \approx K( {{V}_{g1} - {V}_{g2}} )$, which accounts for the nearly linear bipolar tuning observed under antisymmetric gating (Note S2).

### 3 × 3 programmable SrTiO_3_ device array

**Figure 5.**
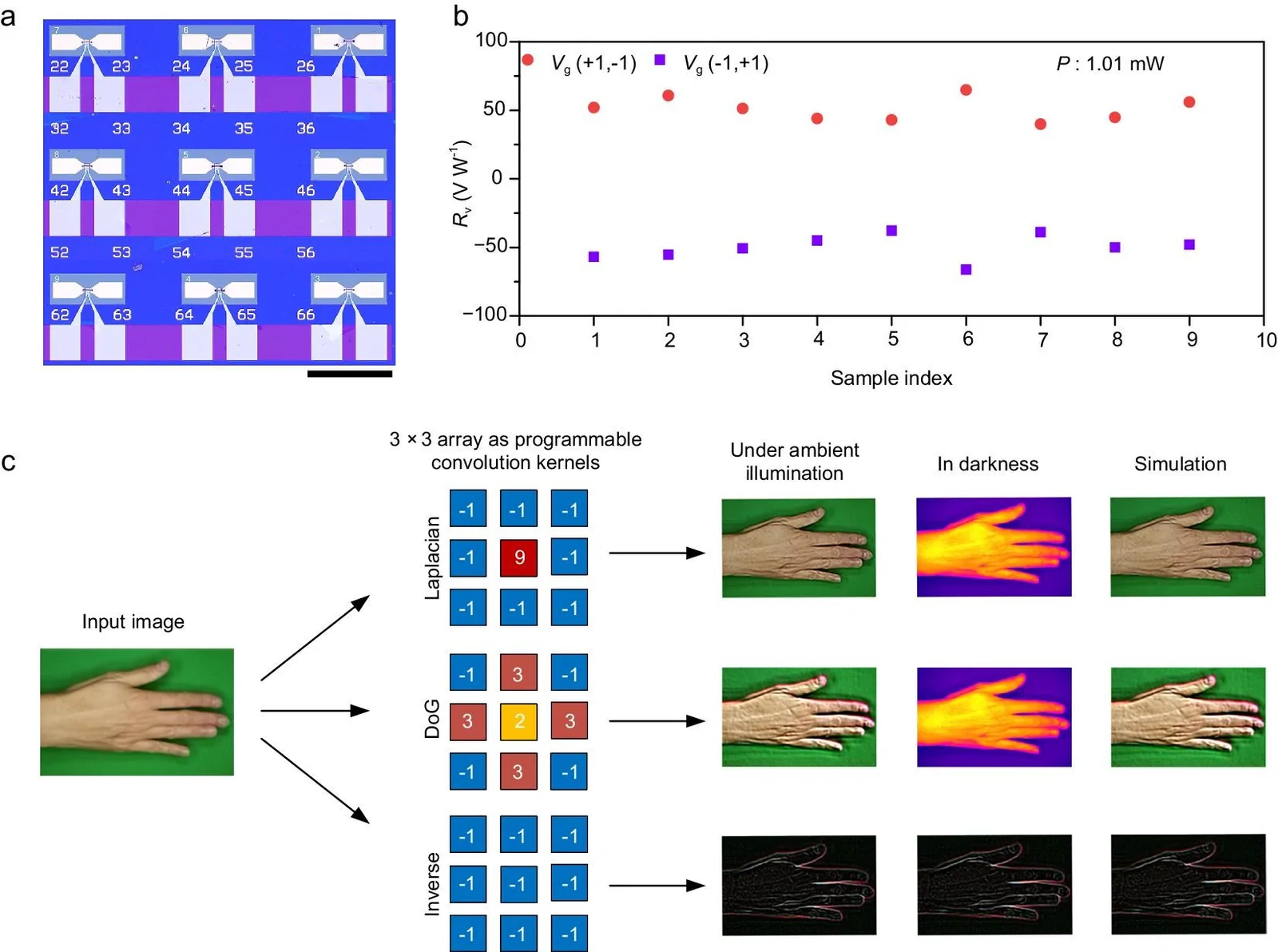
Programmable 3 × 3 SrTiO_3_ detector array and infrared image preprocessing. (a) Optical image of the 3 × 3 suspended SrTiO_3_ device array. Scale bar: 500 μm. (b) Device-to-device responsivity under opposite antisymmetric gate configurations measured under center illumination. (c) Programmable kernel operations enabled by positive and negative PTE responsivity states. Representative Laplacian, DoG, and inverse kernels are mapped onto the programmable responsivity states of the detector array and applied to visible and infrared inputs.

Here, we fabricated a 3 × 3 array of suspended SrTiO_3_ membrane devices, which can be configured as programmable convolutional kernels for infrared image preprocessing. Figure 5a shows the optical image of the array, in which nine suspended SrTiO_3_ membrane devices are integrated on the same substrate. Cross-sectional SEM characterization further confirms the successful formation of suspended nanometer-thick SrTiO_3_ membranes in all nine devices, with thicknesses ranging from 146 to 316 nm (Fig. S13). Time-resolved measurements further show that all array devices exhibit fast microsecond-scale photoresponse, confirming that the rapid temporal dynamics of the suspended nanometer-thick SrTiO_3_ architecture are preserved across the array (Fig. S14). Notably, the thinnest device with a membrane thickness of 146 nm exhibits a response time of ∼5 μs, further supporting the above conclusion that thickness scaling accelerates the PTE response by reducing the active thermal mass.

We then evaluated the device-to-device consistency of the programmable response. As shown in Fig. 5b, all nine devices exhibit positive responsivity under the (±) gate configuration and negative responsivity under the (−/+) gate configuration, demonstrating reproducible polarity switching across the array. Although the responsivity magnitude varies among devices, mainly due to variations in membrane thickness, contact geometry, and local suspended structure, the gate-programmable polarity is consistently preserved. Additional measurements on each array element further confirm reproducible left/right illumination polarity, center-channel dual-gate switching, and stable dark *I*–*V* characteristics before and after repeated gate cycling (Figs S15–S17). These results verify that the programmable SrTiO_3_ PTE response can be extended to an array-level platform, providing the device basis for subsequent infrared image preprocessing and recognition tasks.

### Programmable infrared image preprocessing and recognition

Taking advantage of its wavelength-independent responsivity and linear power response, the 3 × 3 programmable device array was configured as convolutional kernels for infrared image preprocessing in the 0.4–10 μm spectral range. Electrostatic modulation of the Seebeck profile enables kernels such as inverse, difference-of-Gaussian (DoG), and Laplacian, allowing the device to function not only as an imager but also as a programmable preprocessing unit. In low-visibility environments, where visible cameras fail to provide sufficient contrast, human bodies and other warm objects emit strong MIR radiation that can be directly captured and enhanced by the SrTiO_3_ PTE device. Through kernel operations, including noise filtering, edge detection, and contrast amplification, target visibility is significantly improved for subsequent recognition (Fig. 5c).

**Figure 6.**
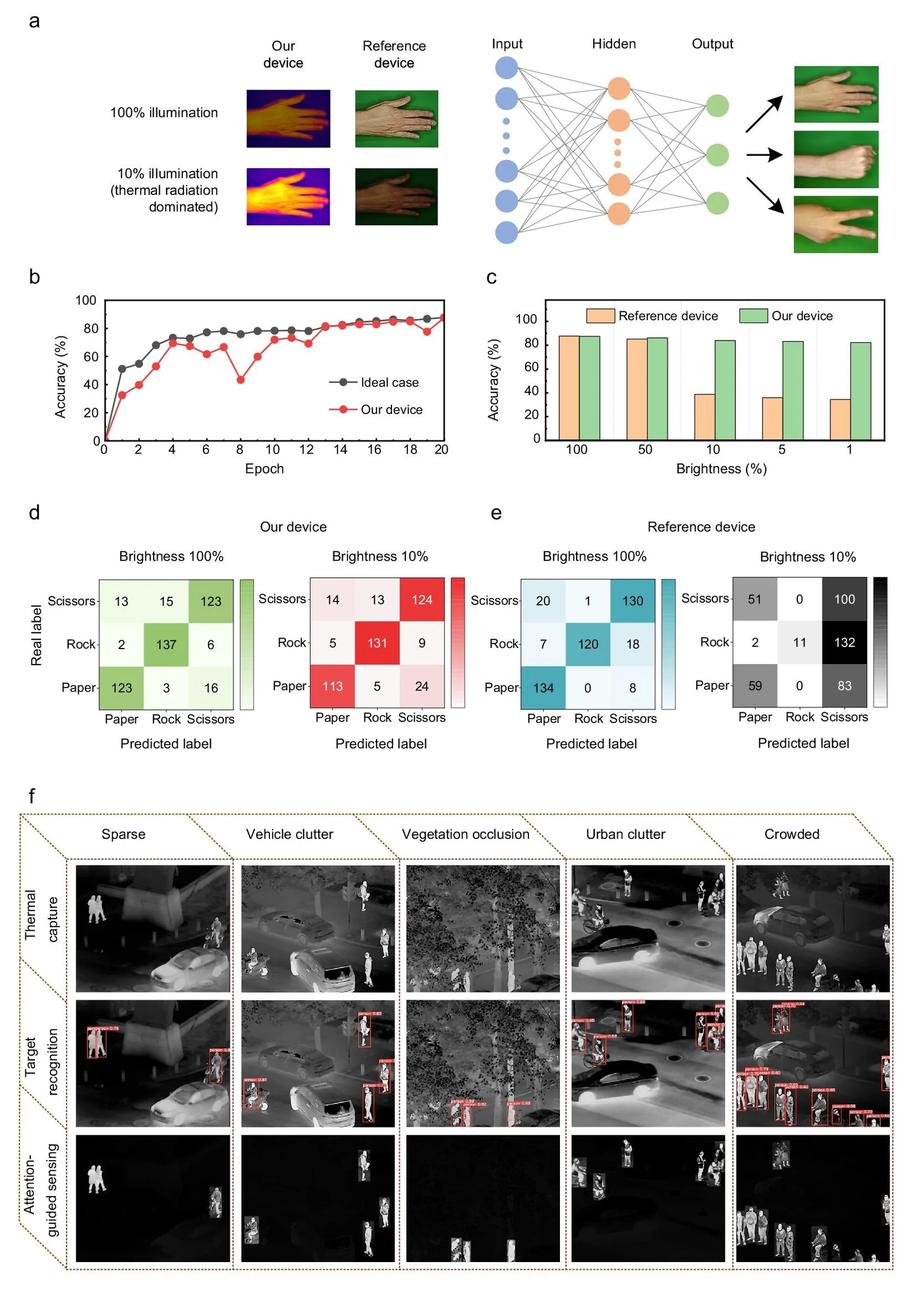
Infrared recognition and attention-guided infrared sensing using programmable SrTiO_3_ PTE detectors. (a) ANN-based gesture-recognition scheme comparing the SrTiO_3_ PTE detector with a visible reference sensor. (b) Training accuracy as a function of epoch. (c) Recognition accuracy under different ambient-brightness levels. (d and e) Confusion matrices at 100% and 10% brightness for the SrTiO_3_ PTE photodetector arrays (d) and reference schemes (e). (f) Dataset-based demonstration of attention-guided infrared recognition in representative thermal scenes, including sparse targets, vehicle clutter, vegetation occlusion, urban clutter, and crowded scenes. The rows show the initial thermal capture, target-recognition results, and recognition-guided responsivity-reconfigured outputs, respectively. Target regions are assigned enhanced responsivity, whereas background regions are assigned suppressed responsivity, enabling target-enhanced infrared sensing in complex scenes.

Beyond image preprocessing, the device exploits its positive and negative multi-level PTE responsivities as reconfigurable weights, enabling their use as reconfigurable weights in an artificial neural network (ANN) framework [4,6,36–39]. As shown in Fig. 6a, we implemented a single-hidden-layer ANN for gesture recognition across visible and infrared spectral ranges, using a conventional visible-only ANN element as a comparison. Under bright illumination, both systems achieve robust recognition of hand gestures, reaching >71% accuracy within only two training epochs (Fig. 6b). However, when the ambient brightness decreases, the visible-domain image input becomes severely degraded and eventually approaches the noise floor, with accuracy dropping below 34% at <1% brightness. In contrast, the SrTiO_3_ PTE device can still capture mid- to long-wave infrared thermal emission from warm targets, which is largely independent of ambient visible illumination. As a result, the device sustains recognition accuracy above 83% even under dark conditions (Fig. 6c). The confusion matrices further highlight this distinction at 10% ambient light, where the SrTiO_3_ PTE-based scheme maintains near-full-brightness recognition performance (Fig. 6d), whereas the visible-domain programmable image sensor suffers from severe misclassification (Fig. 6e).

After demonstrating programmable-kernel infrared preprocessing and downstream image classification, we further demonstrate attention-guided infrared recognition by using the recognition result to reconfigure the pixel-level responsivity of the programmable SrTiO_3_ PTE array. To evaluate this strategy in more complex scenarios, we performed a dataset-based simulation using thermal images from the LLVIP dataset as representative infrared scenes. As shown in Fig. 6f, the first row shows the initial thermal capture under different pedestrian-recognition conditions, including sparse targets, vehicle clutter, vegetation occlusion, urban clutter, and crowded scenes. Target regions were then identified from the thermal images to generate recognition-guided attention masks, as shown in the second row. Based on these masks, pixels corresponding to the target regions were assigned higher gate bias and enhanced responsivity (*V*_g1_ = +1 V, *V*_g2_ = −1 V, *R*_v_ = ∼50.8 V W^−1^), whereas non-target background pixels were assigned lower gate bias and suppressed responsivity (*V*_g1_ = +0.2 V, *V*_g2_ = −0.2 V, *R*_v_ = ∼3.65 V W^−1^). As shown in the third row in Fig. 6f, recognition-guided responsivity reconfiguration selectively enhances target-related thermal signatures while suppressing background interference. This simulation indicates that the programmable SrTiO_3_ PTE array can support recognition-guided pixel-level responsivity reconfiguration.

## CONCLUSION

In conclusion, we have demonstrated a programmable PTE detector based on suspended, nanometer-thick SrTiO_3_ membranes. By reducing the active thermal mass and preserving lateral temperature gradients, the device exhibits broadband, self-powered infrared detection with a response time of 10 μs. Dual-gate modulation of the local Seebeck profile further allows the center-channel photoresponse to be continuously programmed in both amplitude and polarity, yielding a responsivity of ∼50 V W^−1^, and 40 experimentally measured stable response states under fixed infrared illumination. We further extend this concept to a 3 × 3 programmable SrTiO_3_ device array, which supports programmable-kernel infrared image preprocessing. Low-visibility image classification and attention-guided infrared recognition are also demonstrated, with a recognition accuracy of 83% under reduced visible illumination. These results establish nanometer-thick perovskite-oxide thermoelectrics as a viable platform for reconfigurable infrared vision and intelligent sensing.

## METHODS

### The fabrication of SrTiO_3_ PTE devices

The device substrates were fabricated on Si wafers. Pt bottom-gate electrodes were patterned first, followed by deposition of a SiO_2_ insulating layer. Air windows were then opened in the SiO_2_ layer by etching, and Au source–drain electrodes were deposited on top. Thin SrTiO_3_ membranes were prepared from 0.5 at% Nb-doped SrTiO_3_ (100) single crystals by focused ion beam (FIB) milling and lift-out. The membranes were transferred onto the pre-patterned substrates and aligned across the Au source–drain electrodes over the air windows. After transfer, carbon-based adhesive was applied at the peripheral Au/SrTiO_3_ contact edges, and the contact regions were reinforced by FIB-assisted tungsten deposition. The carbon powder and tungsten reinforcement were confined to the contact regions and were not introduced into the suspended SrTiO_3_ channel.

### Structural characterization

The device morphology and suspended structure were characterized by top-view, tilted-view, and cross-sectional SEM using a dual-beam FIB–SEM system (Thermo Fisher, Helios 5 UX). Broad-spectrum (0.4–12 μm) optical absorption measurements were performed on the samples using a Fourier transform infrared spectrometer (Bruker/INVENIO-R).

### Optoelectronic measurements

Electrical and photovoltage measurements were carried out on a probe station under ambient conditions. *I*–*V* characteristics were measured using a source meter (Keithley 2450). For dual-gate measurements, gate voltages were applied using a Keithley 6482 instrument. For steady-state and high-speed photovoltage measurements, the incident light was modulated using a function generator (DG822, RIGOL, 25 MHz). The open-circuit photovoltage signals were amplified using a low-noise voltage preamplifier and then recorded using a PicoScope oscilloscope. For spatial photovoltage mapping, the focused laser spot was scanned across the device while the photovoltage was recorded at each position. Dark voltage noise spectral density was measured using a spectrum analyzer (PDA Fs-Pro).

### Image processing and recognition simulations

For image-processing simulations, representative thermal infrared images were selected from public datasets. Inverse, DoG, and Laplacian kernels were implemented by mapping their weights to the measured positive and negative responsivity states of the programmable SrTiO_3_ PTE array. The corresponding 3 × 3 kernels were applied to the thermal images to obtain preprocessed outputs.

For gesture recognition, the public Rock-Paper-Scissors dataset was used. The RGB images were augmented with a simulated infrared channel to construct visible-infrared multimodal inputs. The Rock-Paper-Scissors image dataset was obtained from the open-source rps-cv project [40]. The dataset contains three categories of hand gestures (rock, paper, and scissors) and was used as a benchmark dataset for evaluating the image classification capability of the proposed system. Different visible-light conditions were simulated by applying an illumination factor to the RGB channels while keeping the infrared channel unchanged. The generated multimodal images were classified using a single-hidden-layer ANN.

For attention-guided infrared recognition, LLVIP thermal images [41] were used as representative infrared scenes. Target-region masks were generated off-chip from the thermal images. Attention-enhanced outputs were reconstructed by assigning measured high- and low-responsivity states to the target and background regions, respectively. All reconstructions were performed off-chip using experimentally measured SrTiO_3_ PTE responsivity values.

## Supplementary Material

nwag499_Supplemental_File

## Data Availability

Data supporting the findings of this study are available within the paper and the Supplementary Data. Further datasets are available from the corresponding author upon request. The code and algorithm in this study are available from the corresponding author upon request.
