# Supplementary material for: Mid-infrared vision sensor based on suspended perovskite oxides down to nanometer scale
Source: Natl Sci Rev. 2026 Aug 17;13(18):nwag499. doi: 10.1093/nsr/nwag499 (PMC13591642; doi:10.1093/nsr/nwag499)
Supplement: Supplementary file 1 [file nwag499_supplemental_file.pdf]

Supplementary Information for

Mid-infrared vision sensor based on Suspended Perovskite Oxides Down to Nanometer Scale

Haozhe Li[1,2,†], Haoran Mu[2,3,†,*], Wenzhi Yu[4,†], Yang Zhou[2], Kaiwen Gong[1,2], Yun Li[5], Jian Xian He[2], Chen Chen[6], Jinkui Zhao[2,6,7], Daohua Zhang[8], Xiaoxue Song[9], Yuan Yang[1,*], Fang Wang[10,*], Guanyu Zhang[2,7] & Shenghuang Lin[2,*]

[1] School of Integrated Circuits, Xi'an University of Technology, Xi'an 710048, China

[2] Songshan Lake Materials Laboratory, Dongguan 523808, China.

[3] School of Microelectronics Science and Technology, Sun Yat-sen University, Zhuhai, Guangdong 519082, China

[4] Siscan Technology Co., Ltd. Suzhou, Jiangsu 215100, China

[5] Wuhan University of Technology, China

[6] School of Physical Sciences, Great Bay University, Dongguan 523000, China

[7] Institute of Physics, Chinese Academy of Sciences, Beijing, China

[8] Shenzhen Pinghu Laboratory, National Wide Bandgap Semiconductor Technology Innovation Center (Shenzhen), 518111, China

[9] Jiangsu Institute of Advanced Semiconductors LTD

[10] Shanghai Institute of Technical Physics, Chinese Academy of Sciences

[†] These authors contributed equally.

[*] Corresponding authors. E-mails: linshenghuang@sslab.org.cn (Shenghuang Lin); muhr3@mail.sysu.edu.cn (Haoran Mu); Yangyuan@xaut.edu.cn (Yuan Yang); fwang@mail.sitp.ac.cn (Fang Wang).

Contents

Figure S1. Fabrication process of the suspended dual-gate $SrTiO_3$ PTE device 1

Figure S2. Structural verification of the suspended $SrTiO_3$ membrane 3

Figure S3. XRD 2θ scan of 0.5% Nb-doped $SrTiO_3$ 4

Figure S4. Power-dependent time-resolved photoresponse without gate modulation .. 5

Figure S5. Noise, NEP and specific detectivity without gate modulation 6

Figure S6. Optical spot-size calibration at the sample plane 7

Figure S7. Power-dependent time-resolved photoresponse under dual-gate modulation 9

Figure S8. Power-dependent photoresponse under dual-gate modulation 10

Figure S9. Noise, NEP and specific detectivity under dual-gate modulation 11

Figure S10. Raw gate-sweep data for dual-gate photovoltage mapping 12

Figure S11. Capacitance–frequency measurement of the suspended $SrTiO_3$ device for electrical RC analysis 13

Figure S12. Gate-programmed spatial photovoltage response and PTE mechanism . 14

Figure S13. Thickness characterization of representative suspended $SrTiO_3$ membrane devices 15

Figure S14. Device-to-device variation in response time and its thickness dependence under 4 μm illumination 16

Figure S15. Device-to-device reproducibility of photothermoelectric response and dual-gate cycling stability for Devices 1–3 18

Figure S16. Device-to-device reproducibility of photothermoelectric response and dual-gate cycling stability for Devices 4–6 20

Figure S17. Device-to-device reproducibility of photothermoelectric response and dual-gate cycling stability for Devices 7–9 22

Table S1. Performance comparison of infrared detectors based on different materials and operating mechanisms 24

Supplementary Note 1. Mechanistic Interpretation of Thin-Film $SrTiO_3$ for Enhanced Photothermoelectric Response and Programmability 25

Supplementary Note 2. Linear approximation of dual-gate-programmed PTE responsivity

....27

Supplementary Note 3. Noise-limited estimation of resolvable programmable photovoltage states in $SrTiO_3$ PTE detectors

....28

Supplementary References..29

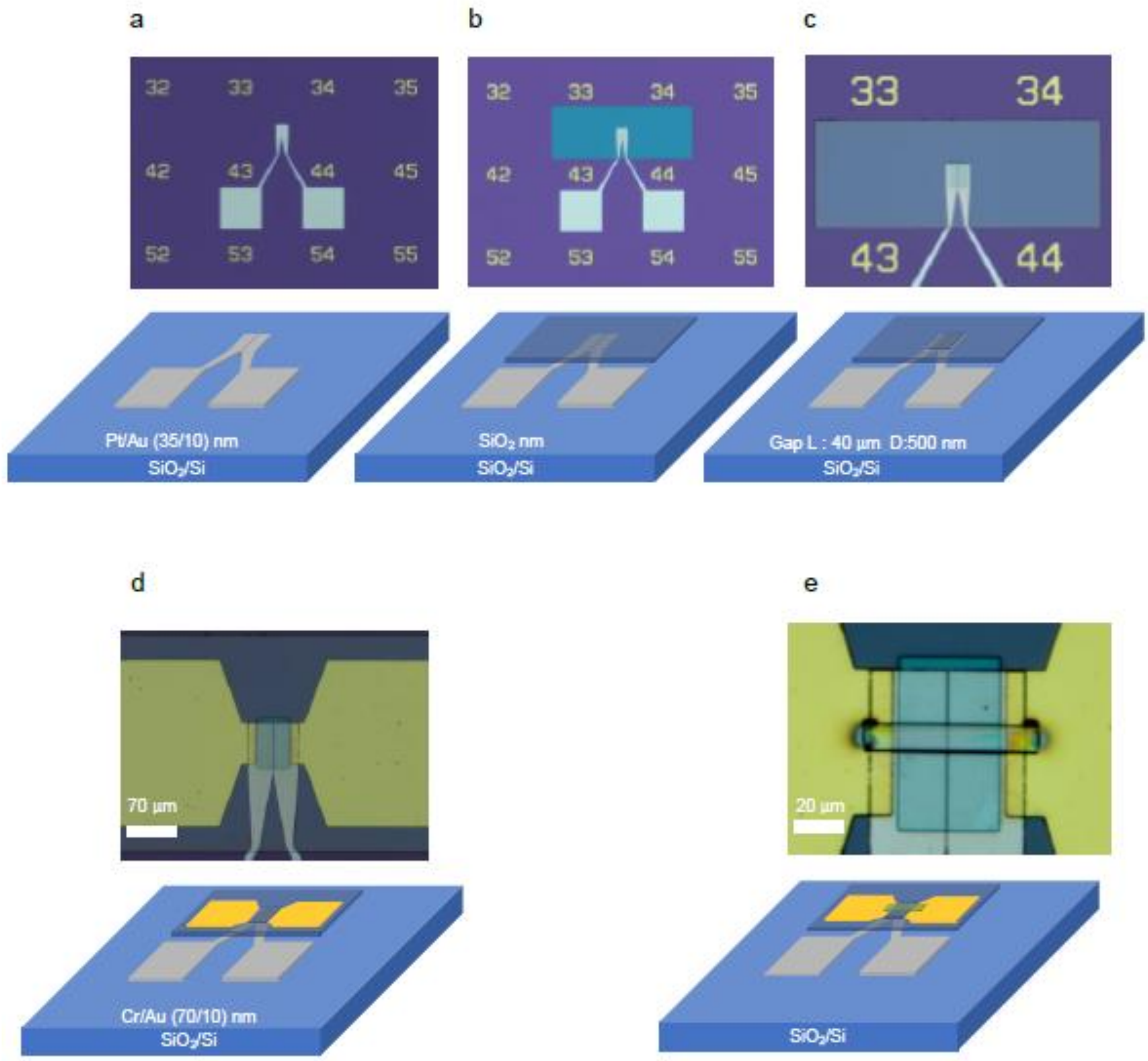

Figure S1. Fabrication process of the suspended dual-gate $SrTiO_3$ PTE device. (a) Split bottom-gate electrodes were patterned on a SiO_2/Si substrate by electron-beam lithography and metal deposition. (b) A 650-nm-thick SiO_2 insulating layer was deposited by atomic layer deposition. (c) A rectangular air window was opened above the split bottom gates by photolithography and HF vapor etching. (d) Cr/Au source–drain electrodes were patterned by electron-beam lithography and metal deposition. (e) A FIB-prepared $SrTiO_3$ membrane was transferred onto the pre-patterned substrate to form a suspended channel over the air window.

The device fabrication process is shown in Fig. S1. Split Pt/Au bottom gates (35/10 nm) were first patterned on a 285-nm-thick SiO_2/Si substrate, followed by deposition of a

650-nm-thick ALD SiO_2. The rectangular air window of 40 × 70 × 0.5 μm^3 was then defined above the split gates by photolithography and HF vapor etching. Cr/Au source-drain electrodes (10/70 nm) subsequently patterned on the substrate. Separately, a $SrTiO_3$ membrane with dimensions of 70 × 9 × 0.2 μm^3 was cut and released from a 0.5 at% Nb-doped $SrTiO_3$ (100) single crystal by FIB milling. A membrane was transferred across the air window as the suspended channel. Considering that the source–drain electrode spacing was is ~ 42 μm and the width of the membrane is ~ 9 μm, the geometrical active channel area is ~ 378 μm^2.

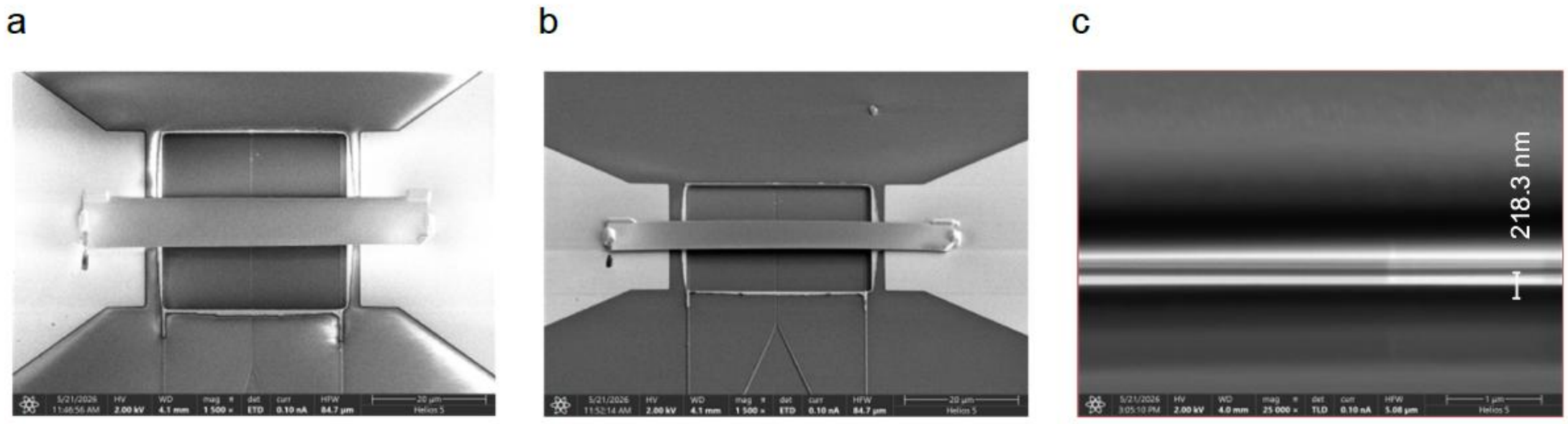

Figure S2. Structural verification of the suspended $SrTiO_3$ membrane. (a) Top-view SEM image of another device. (b) SEM image acquired at a 52° stage tilt, showing the $SrTiO_3$ membrane bridging the source–drain electrodes across the etched air window. (c) Cross-sectional SEM image of the same sample, showing the suspended $SrTiO_3$ membrane above the air-gap region. The tilted-view and cross-sectional SEM images confirm that the suspended $SrTiO_3$ membrane remains free-standing without obvious bending, collapse, or contact with the underlying substrate.

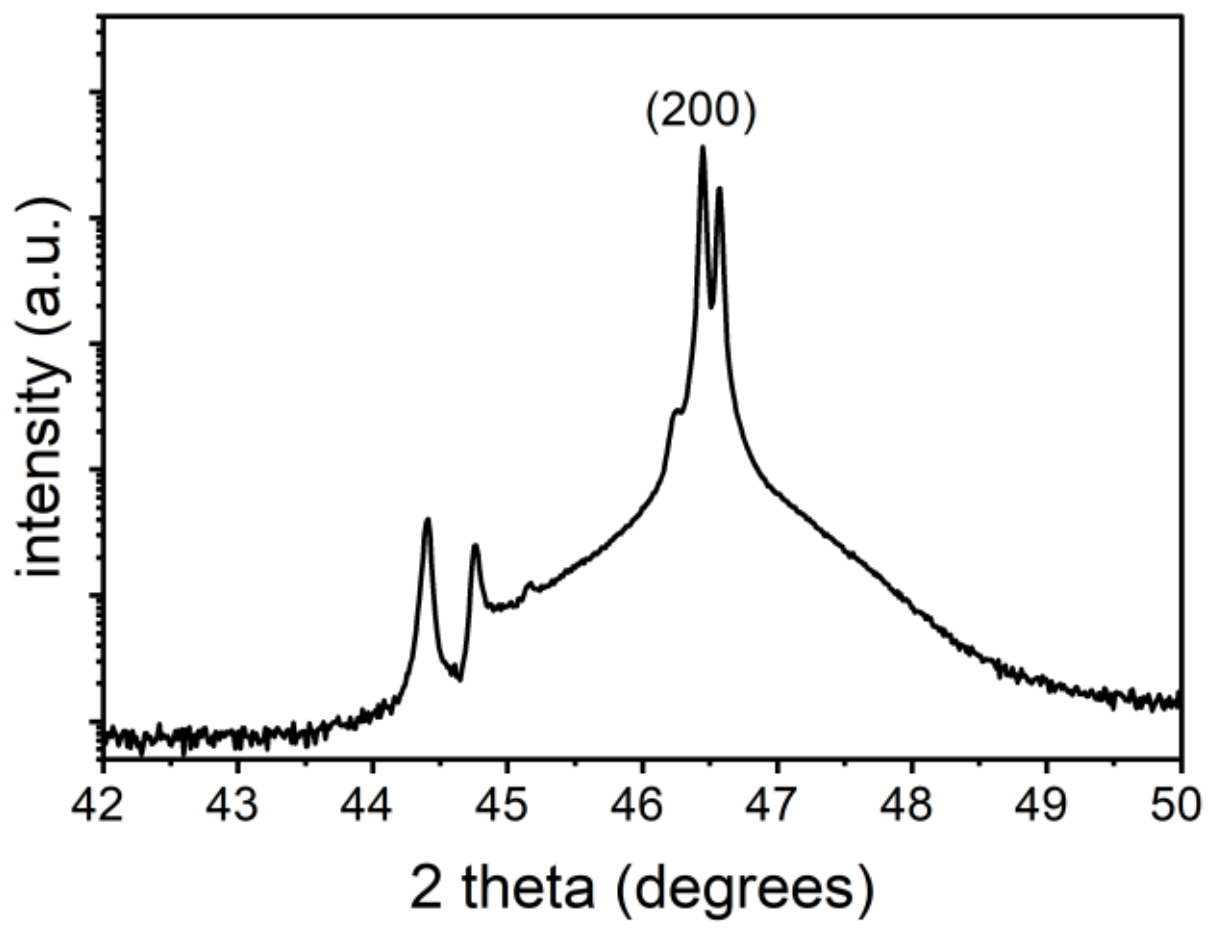

Figure S3. XRD 2θ scan spectrum of 0.5% Nb-doped $SrTiO_3$.

As demonstrated in Fig. S3, the XRD pattern of 0.5% Nb: $SrTiO_3$ displays a distinct (200) diffraction peak at $2\theta = 46.3$, exhibiting a symmetrical profile and the absence of discernible impurity peaks. This observation signifies the elevated phase purity of the cubic perovskite structure (space group Pm-3m). In comparison with undoped $SrTiO_3$, this peak demonstrates a shift of 0.1 – 0.2 towards lower angles, which corresponds to a lattice expansion rate of approximately 0.2%. This phenomenon is attributed to the substitution of Ti^{4+} (r = 0.605 Å) by Nb^{5+} (r = 0.64 Å) and concomitant oxygen vacancy formation.

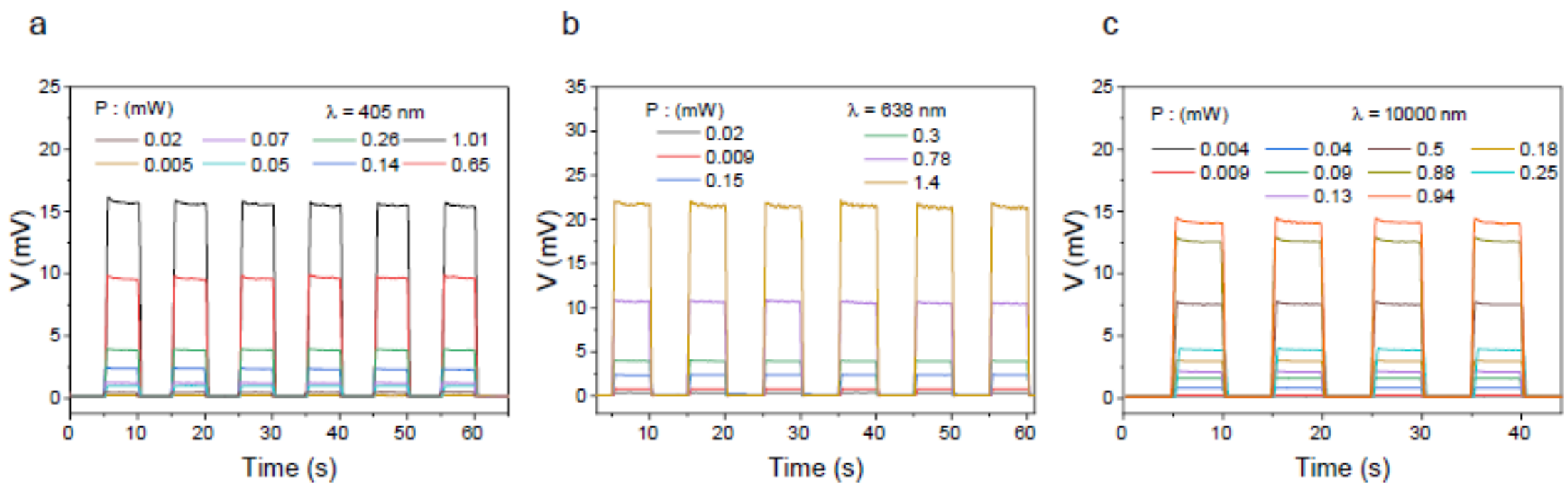

Figure S4. Power-dependent time-resolved photoresponse without gate modulation. Time-resolved photoresponse measured at different incident powers under illumination wavelengths of (a) 405 nm, (b) 638 nm and (c) 10.5 μm. The measurements were performed without gate modulation.

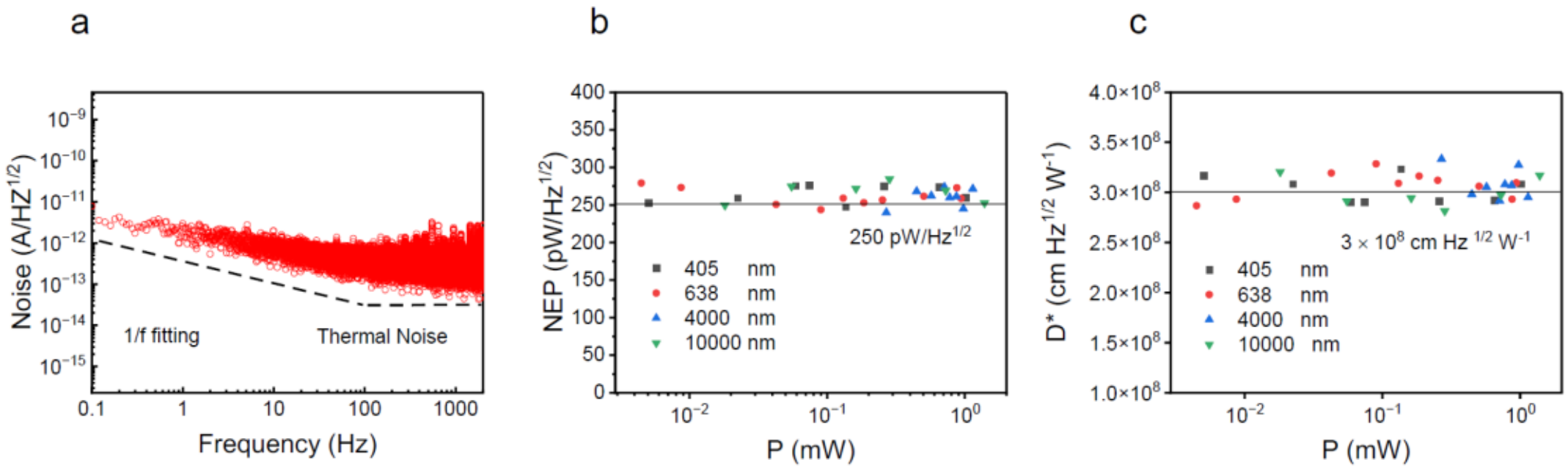

Figure S5. Noise, NEP, and specific detectivity of the suspended $SrTiO_3$ PTE detector without gate modulation. (a) Responsivity versus optical power at multiple wavelengths. (b) Calculated specific detectivity (D*) of the PTE photothermal detector across the visible to mid-infrared spectral range. (c) Noise Equivalent Power (NEP) calculated for mid-infrared and visible spectral regions.

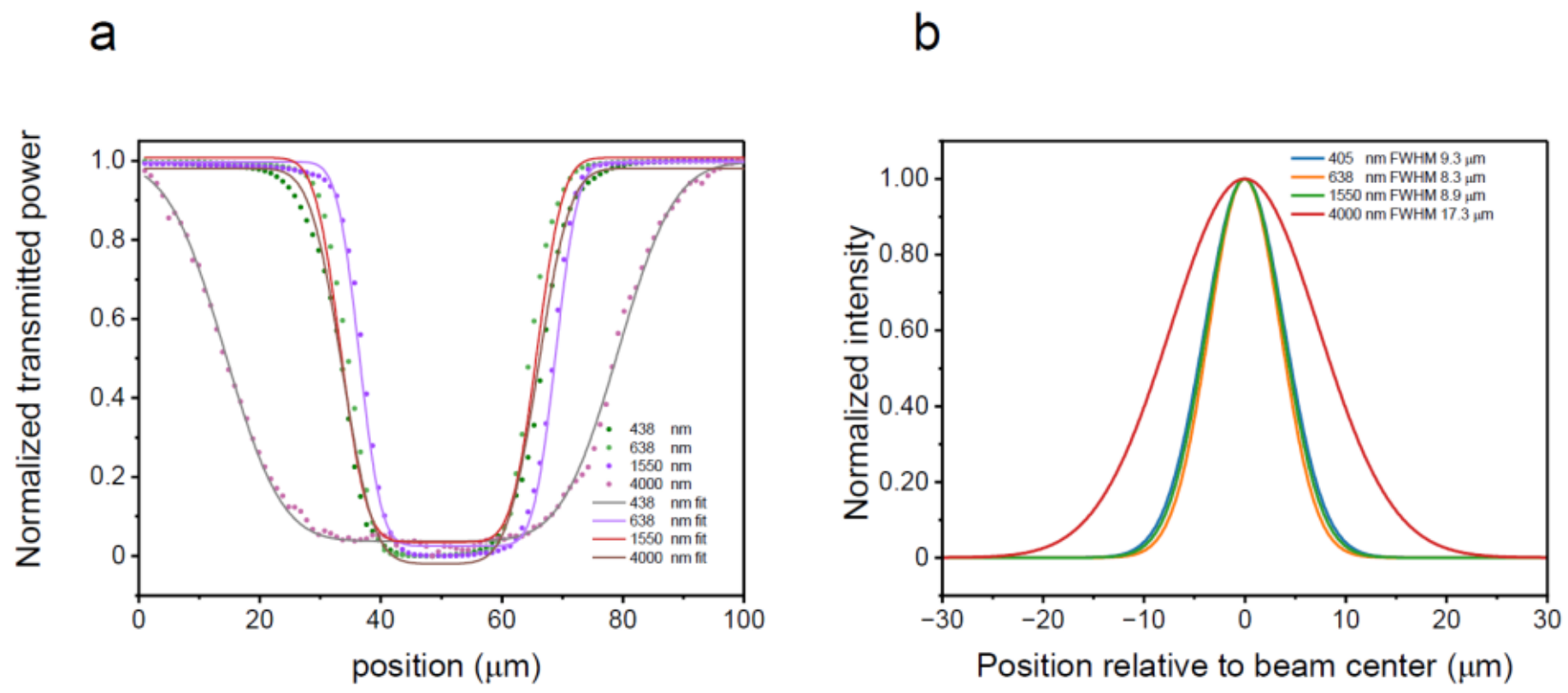

Figure S6. Optical spot-size calibration at the sample plane. (a) Normalized edge-occlusion scan profiles measured at 405, 638, 1550 nm and 4 μm. The data were fitted using a double-edge error-function model to extract the spot diameters at the sample plane. (b) Reconstructed one-dimensional Gaussian beam profiles based on the fitted parameters. The extracted FWHM diameters are approximately 9.3, 8.3, 8.9 and 17.3 μm, corresponding to $1/e^2$ diameters of approximately 15.8, 14.2, 15.1 and 29.4 μm for 405, 638, 1550 and 4000 nm, respectively.

The optical spot size at each wavelength was calibrated at the sample plane using an edge-occlusion scan under the same focusing conditions as those used for photoresponse measurements. A calibrated opaque tip was translated across the focused beam, and the transmitted optical power was recorded using calibrated power sensors. The optical power was measured using Thorlabs S130VC sensors for 405 and 638 nm, an S132C sensor for 1550 nm, and an S401C sensor for 4 μm. The 405, 638, and 1550 nm beams were focused using a 50× objective, whereas the 4 μm beam was focused using a 20× objective.

For a Gaussian beam, the one-dimensional intensity profile is $I(x) = I_0 \exp\left[-\frac{(x-x_0)^2}{2\sigma^2}\right]$, where I_0 is the peak intensity, x_0 is the beam centre and σ is the Gaussian standard deviation. The edge-occlusion trace follows an error-function dependence; therefore, the finite-width tip was fitted using a double-edge error-function model. The fitted beam-width parameter was used to calculate the FWHM diameter, $D_{\mathrm{FWHM}} =$

$2\sqrt{2\ln 2}\sigma \approx 2.355\sigma$; $D_{1/e^2} \approx 4\sigma$. The extracted FWHM diameters are 9.3, 8.3, 8.9, and 17.3 μm for 405, 638, 1550 nm, and 4 μm, respectively, corresponding to ($1/e^2$) diameters of 15.8, 14.2, 15.1, and 29.4 μm. The ($1/e^2$) diameter was used to calculate the effective beam area for power-density and responsivity analyses. For focused-beam measurements, the effective optical power was determined from the measured incident power, the calibrated Gaussian beam profile, and the beam–channel overlap at the sample plane.

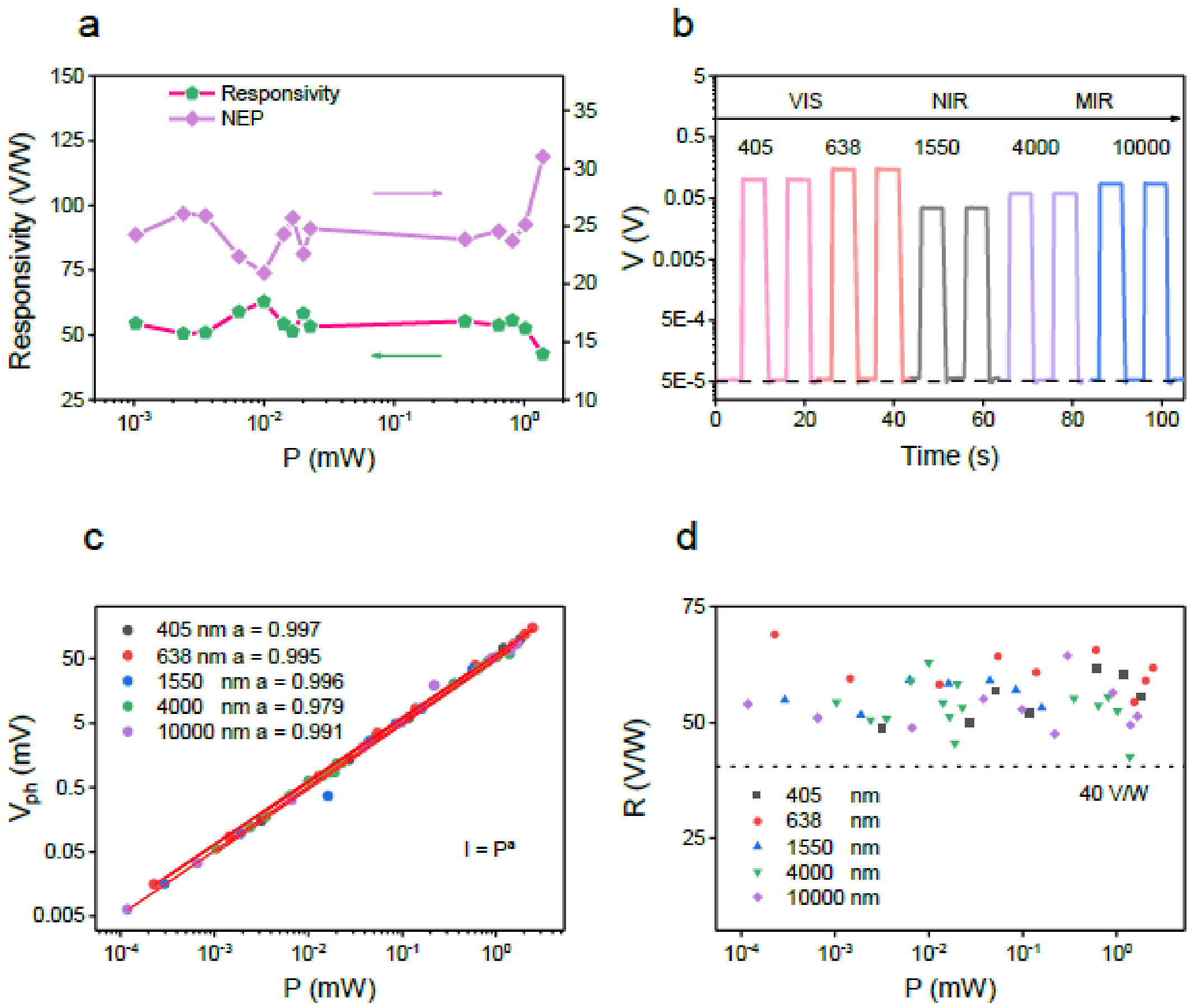

Figure S7. (a-d) Time-resolved photoresponse with different incident light power under various wavelength of 0.4 μm (a), 0.6 μm (b), 1550nm(c), and 10.5 μm (d) (V_{g1} = 1 V, V_{g2} = -1 V).

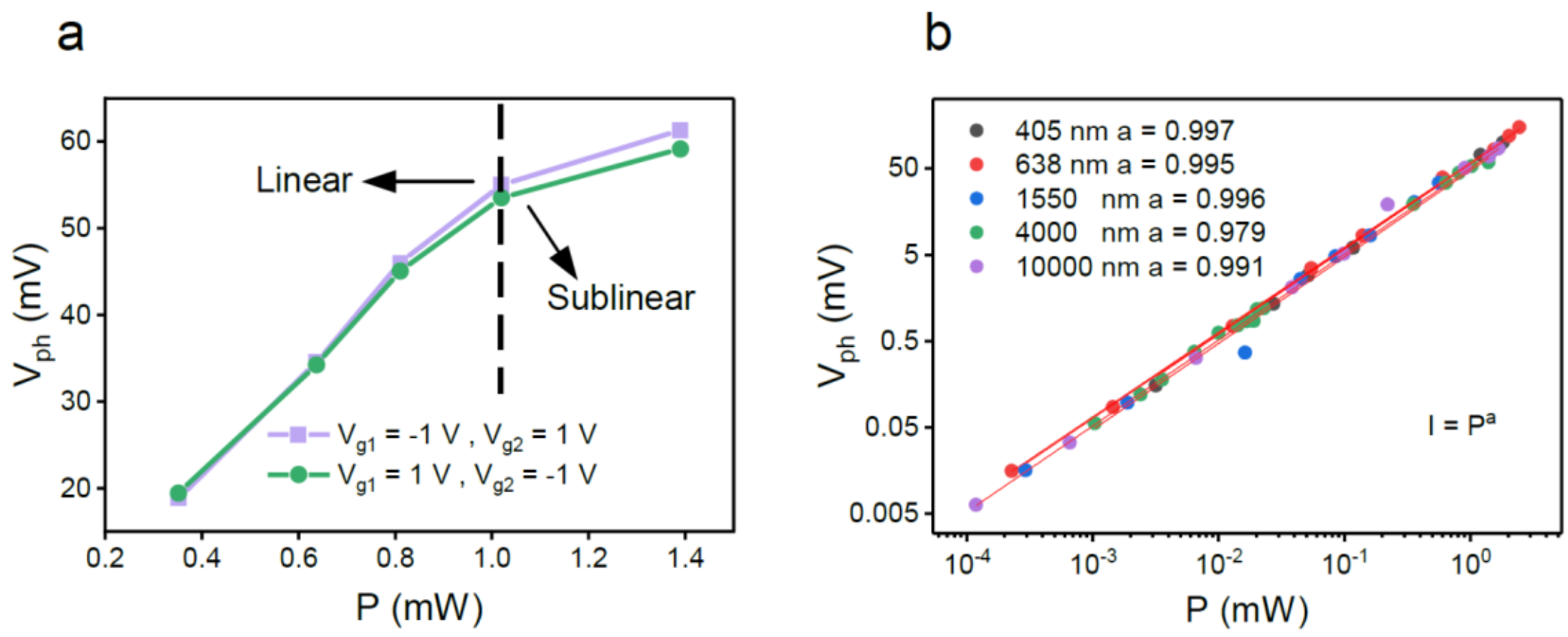

Figure S8. Power-dependent photoresponse under dual-gate modulation. (a and b) Photovoltage and voltage responsivity as a function of incident optical power under programmable dual-gate operation. The wavelength-dependent measurements include 0.4, 0.6, 1.5, 4 and 10.5 μm illumination. Solid lines indicate power-law fits used to evaluate the linearity of the photovoltage response.

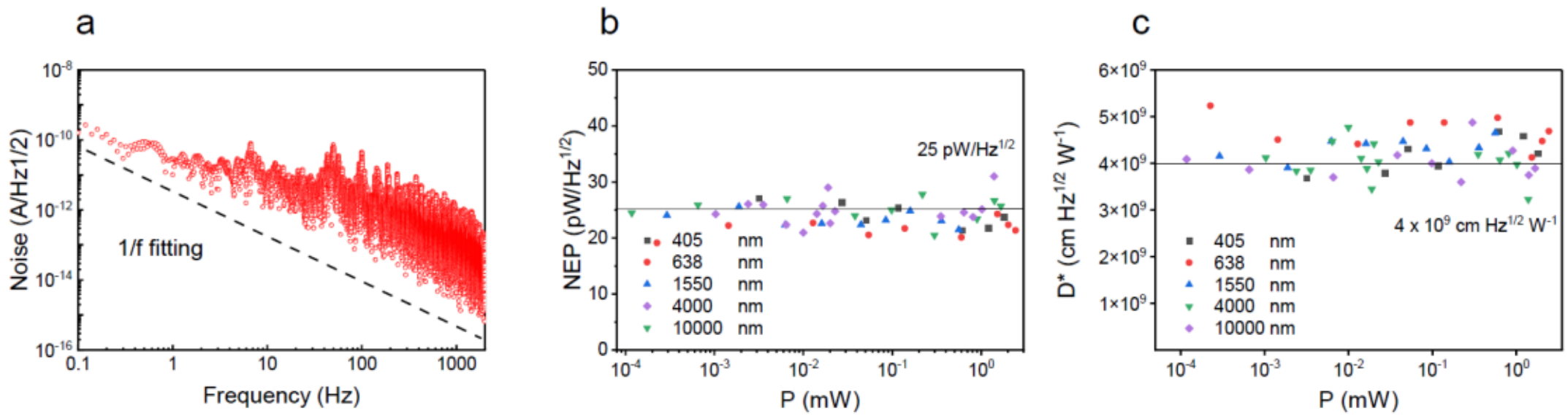

Figure S9. Noise, NEP, and specific detectivity of the dual-gated $SrTiO_3$ PTE detector. (a) Noise current data measured using a lock-in amplifier through PTE devices. (b) Specific detection rate (D*) of PTE photothermal detectors in the visible to mid-infrared spectral range. (c) Noise equivalent power (NEP) calculated for the mid-infrared and visible spectral regions.

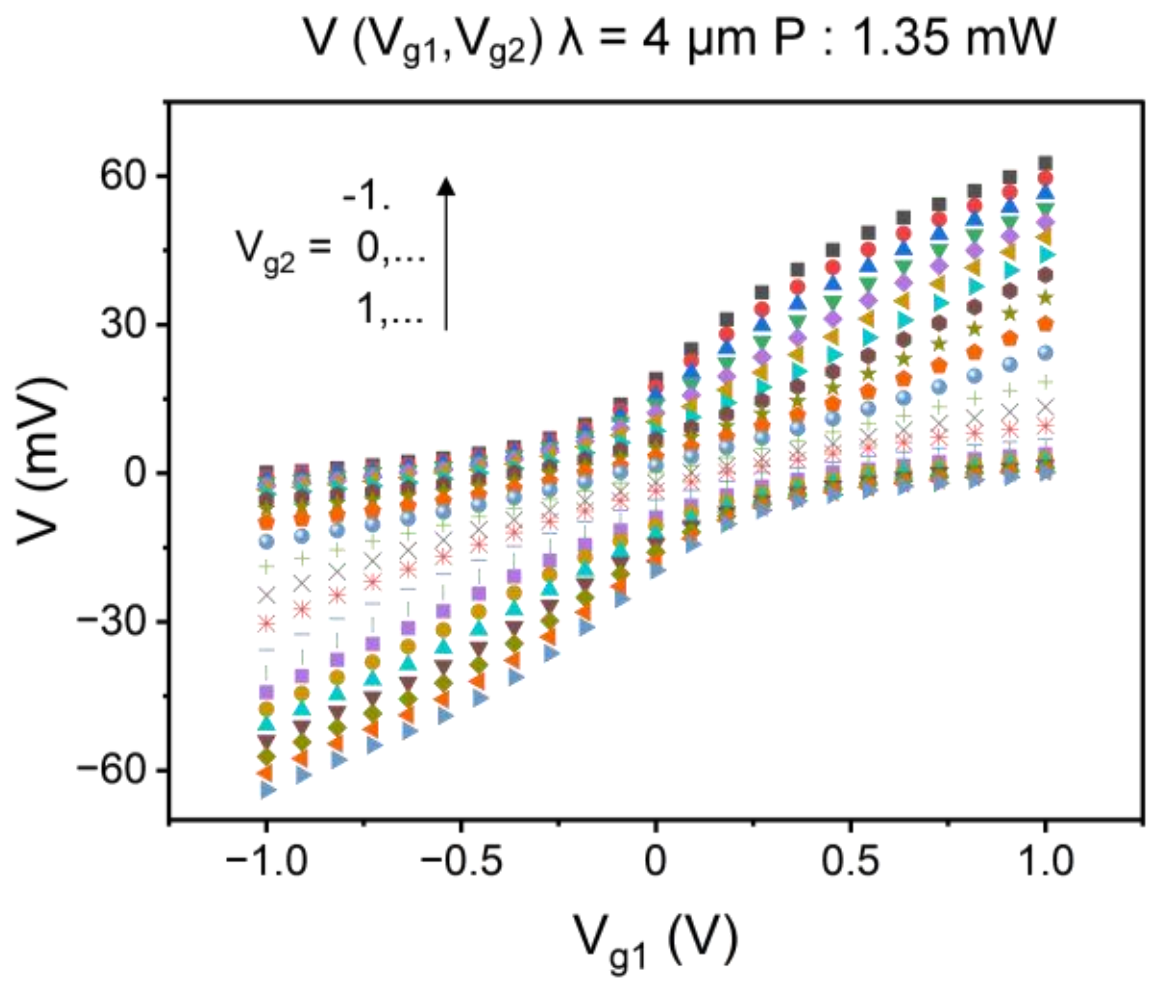

Figure S10. Raw gate-sweep data for dual-gate photovoltage mapping.

Photovoltage measured as a function of V_{g1} while V_{g2} was stepped from -1 to +1 V under 4 μm illumination with a fixed incident power of 1.36 mW. These raw gate-sweep curves were used to construct the two-dimensional V_{g1} - V_{g2} photovoltage map shown in Fig. 3g.

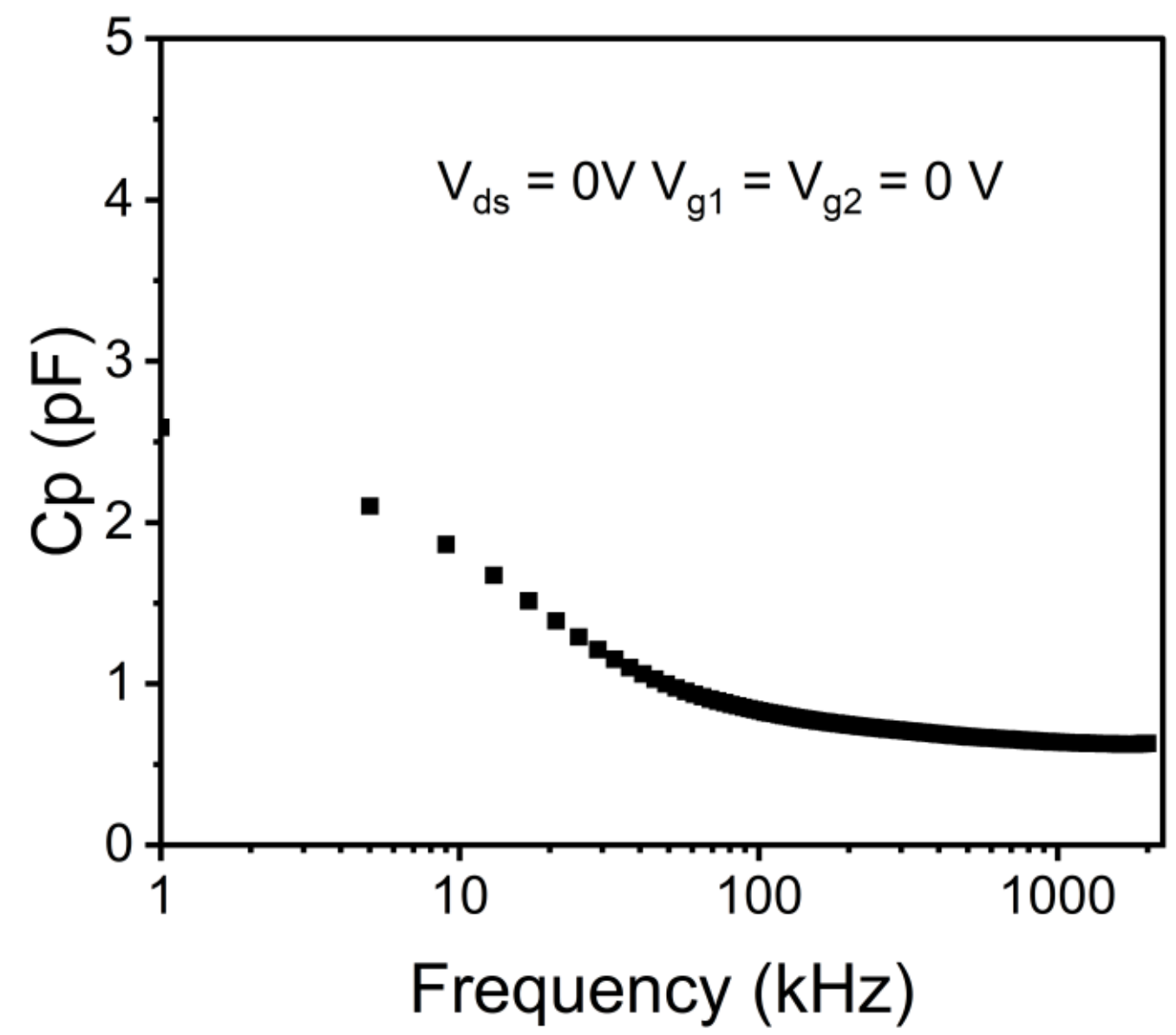

Figure S11. Capacitance–frequency measurement of the suspended $SrTiO_3$ device for electrical RC analysis. Capacitance-frequency characteristics were measured using a Semiconductor Parameter analyser (Keysight B1500A) under dark conditions. The capacitance remains on the pF scale over the measured frequency range, together with the channel resistance extracted from the dark I-V_{ds} curve, to estimate the electrical RC time constant.

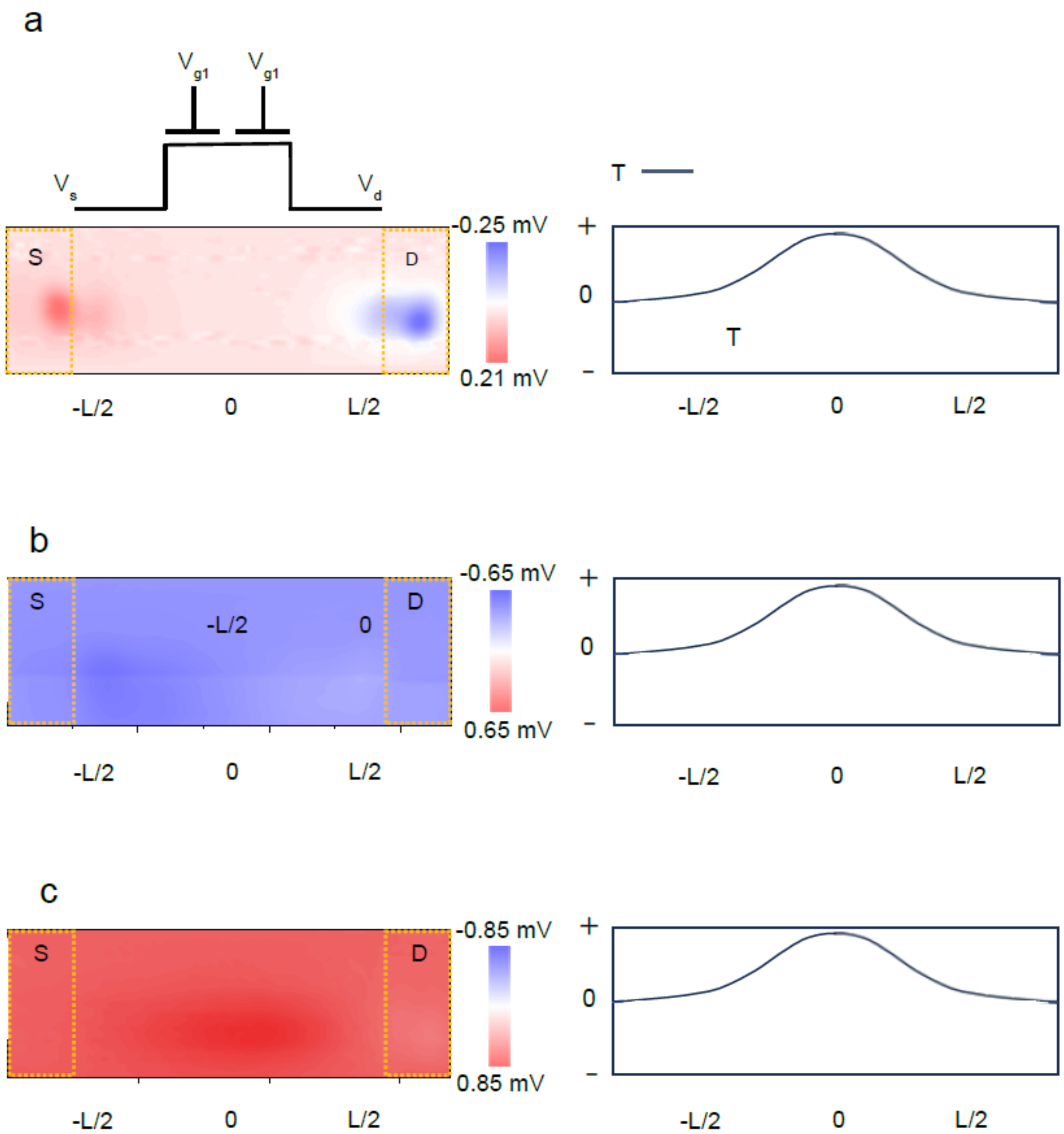

Figure S12. Gate-programmed spatial photovoltage response and PTE mechanism. Schematic and spatially resolved photovoltage response under three gate-programming conditions. The horizontal coordinate x denotes the position along the suspended channel. T(x) represents the illumination-induced temperature profile, and S(x) represents the programmed Seebeck-coefficient distribution. At $V_{g1} = V_{g2} = 0$ V, centre illumination produces nearly cancelling thermoelectric contributions from the two channel halves. Under antisymmetric gate bias, $V_{g1} = -1$ V, $V_{g2} = 1$ V or $V_{g1} = 1$ V, $V_{g2} = -1$ V, the Seebeck profile becomes asymmetric, generating a finite centre-illumination photovoltage with gate-controllable polarity. These spatially resolved measurements support a gate-programmed PTE mechanism. Without gate modulation, opposite

thermoelectric contributions from the two channel halves approximately cancel under centre illumination. Under antisymmetric gate biasing, the Seebeck profile becomes asymmetric, so centre illumination produces a finite photovoltage with reversible polarity. Together with the broadband response from the visible to mid-infrared range, these spatial and gate-polarity-dependent signatures indicate that the zero-bias response is PTE-dominated.

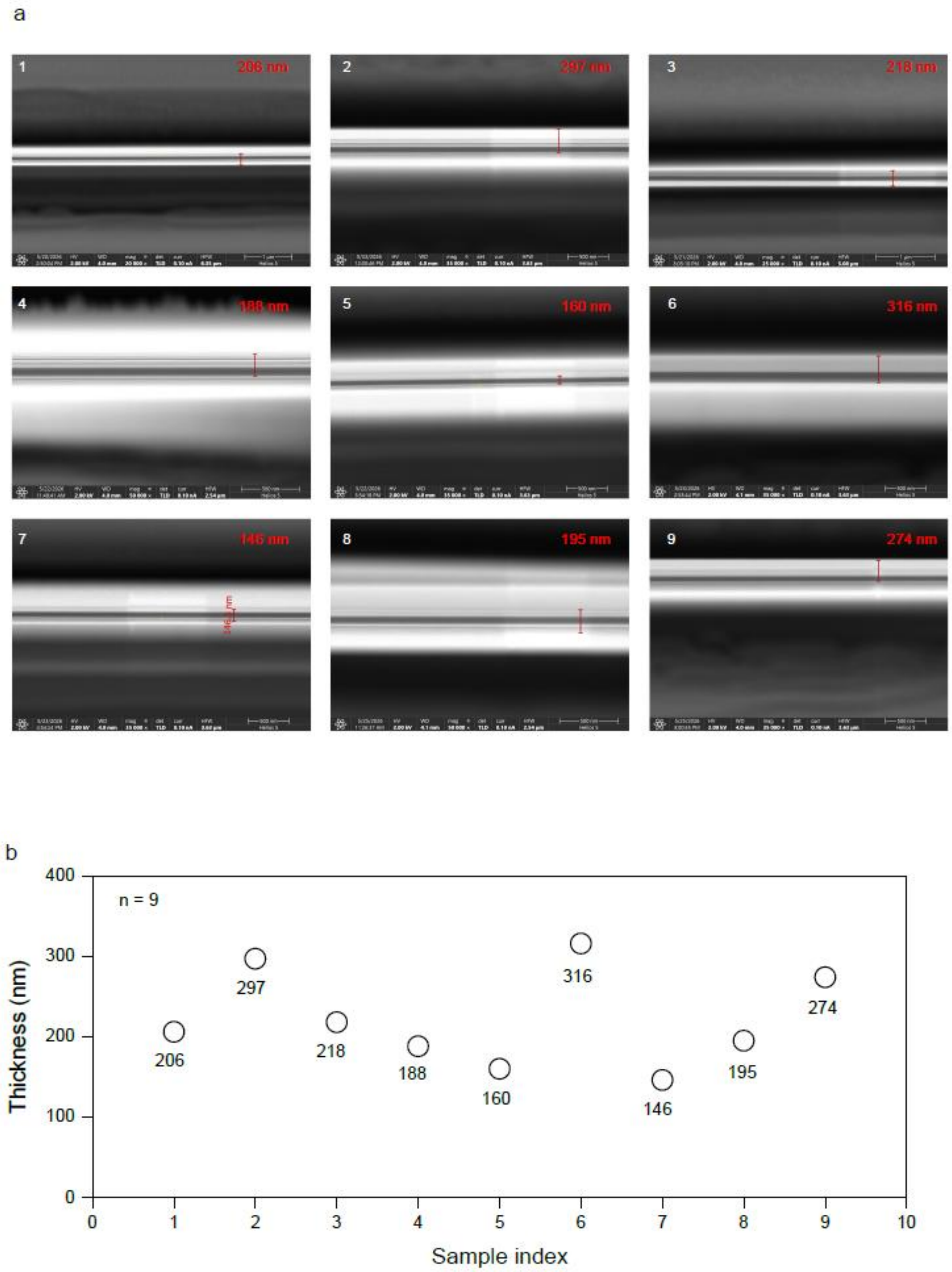

Figure S13. Thickness characterization of representative suspended $SrTiO_3$ membrane devices. Thickness measurements of representative suspended $SrTiO_3$ membranes used to evaluate the device-to-device variation and the thickness-dependent photoresponse dynamics.

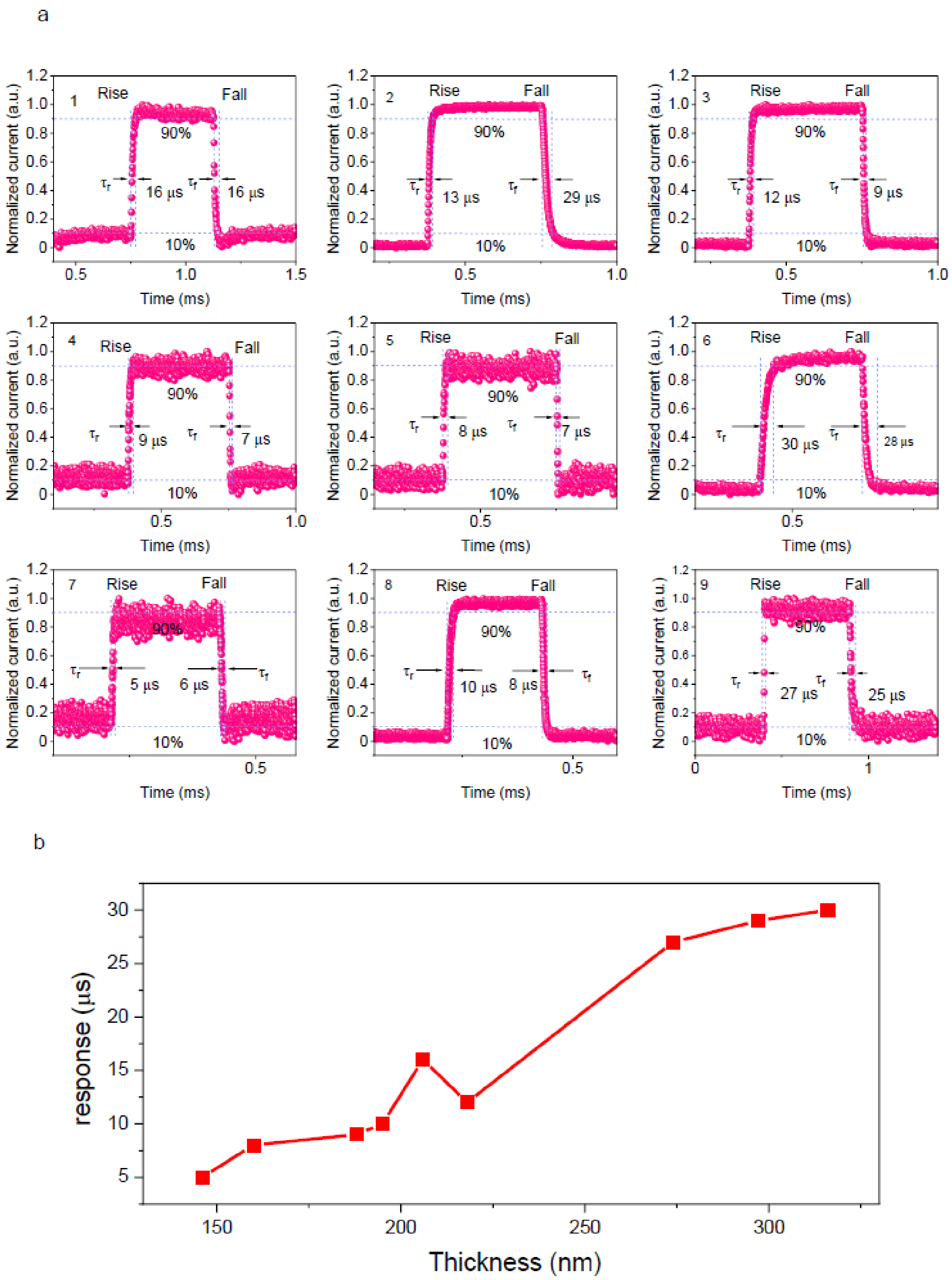

Figure S14. Device-to-device variation in response time and its thickness dependence under 4 μm illumination. All measurements were performed under 4 μm illumination at zero source-drain bias. (a) Response times of nine suspended $SrTiO_3$ devices. (b) Correlation between membrane thickness and response time, showing that thinner membranes generally exhibit faster responses. Representative membrane thicknesses

of 146, 206 and 367 nm correspond to response times of approximately 6, 16 and 30 μs, respectively.

All measurements were performed under 4 μm illumination at V_{ds} = 0 V. The device-to-device statistics show a consistent trend in which thinner suspended membranes exhibit faster photoresponse. This behaviour supports the role of reduced thermal mass in accelerating the PTE response, while the absolute response time remains governed by the full suspended-device thermal circuit.

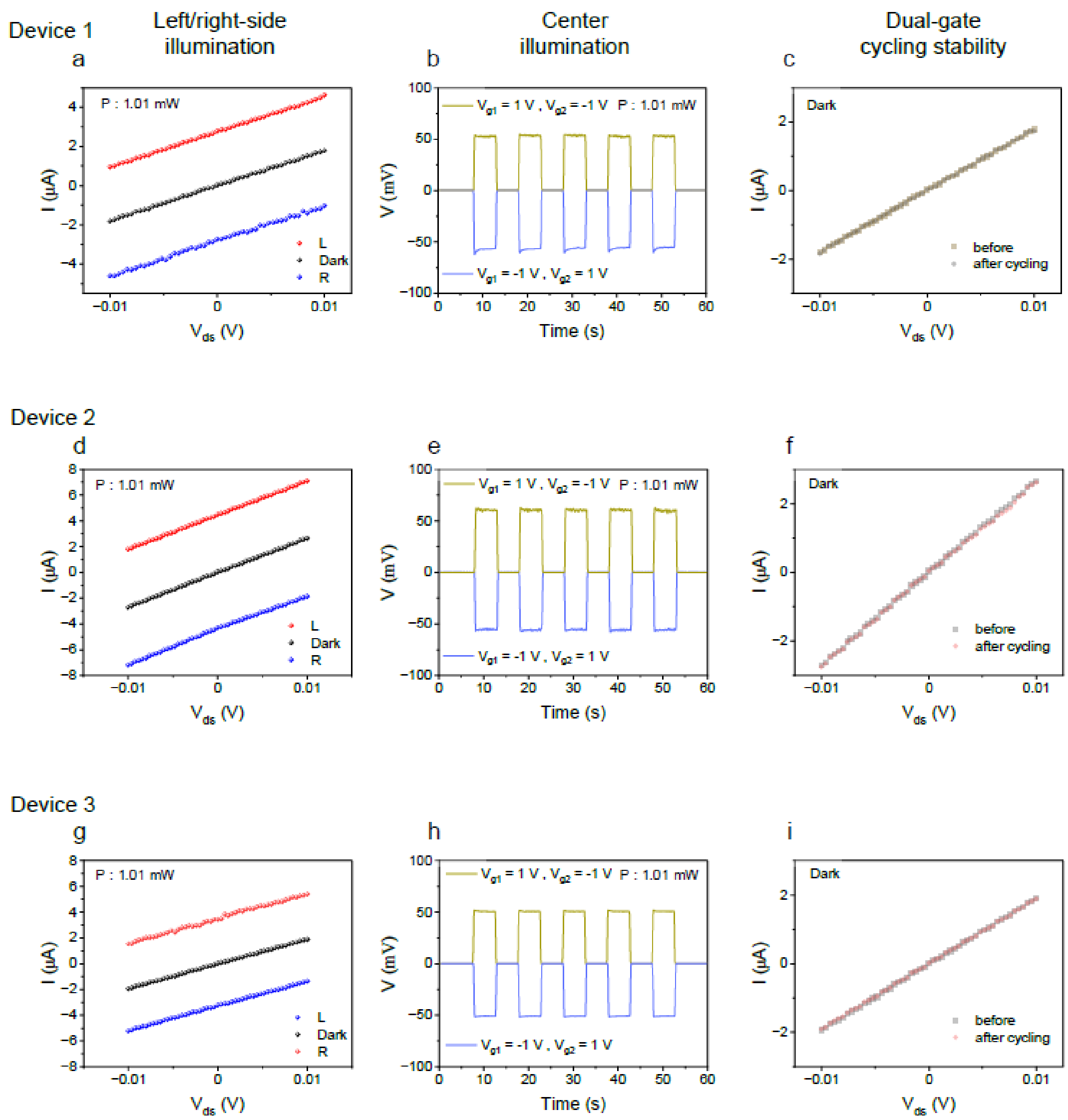

Figure S15. Device-to-device reproducibility of photothermoelectric response and dual-gate cycling stability for Devices 1–3. (a,d,g) Source-drain I-V characteristics of Devices 1-3 measured in the dark and under 4 μm illumination with an incident power of 1.01 mW focused near the left or right contact region. The opposite horizontal shifts under left- and right-side illumination indicate polarity-reversible PTE response. (b,e,h) Time-resolved photovoltage responses measured with the 4 μm laser focused at the channel centre under antisymmetric dual-gate programming. The output polarity reverses when the gate polarity is switched between V_{g1} = -1 V, V_{g2} = 1 V and V_{g1} = 1 V, V_{g2} = -1 V. These measurements were performed at Vds = 0 V. (c,f,i) Dark characteristics measured before and after repeated dual-gate cycling. The nearly overlapping curves indicate stable channel conductance and contact behaviour after

programming cycles. (a,d,g) Source-drain I-V characteristics of Devices 1-3 measured in the dark and under 4 μm illumination with an incident power of 1.01 mW focused near the left or right contact region. The opposite horizontal shifts under left- and right-side illumination indicate polarity-reversible PTE response. (b,e,h) Centre-illumination photovoltage transients measured at V_{ds} = 0 V under antisymmetric dual-gate programming. The photovoltage polarity reverses when the gate polarity is reversed. (c,f,i) Dark I-V characteristics measured before and after repeated dual-gate cycling, showing stable channel conductance and contact behaviour.

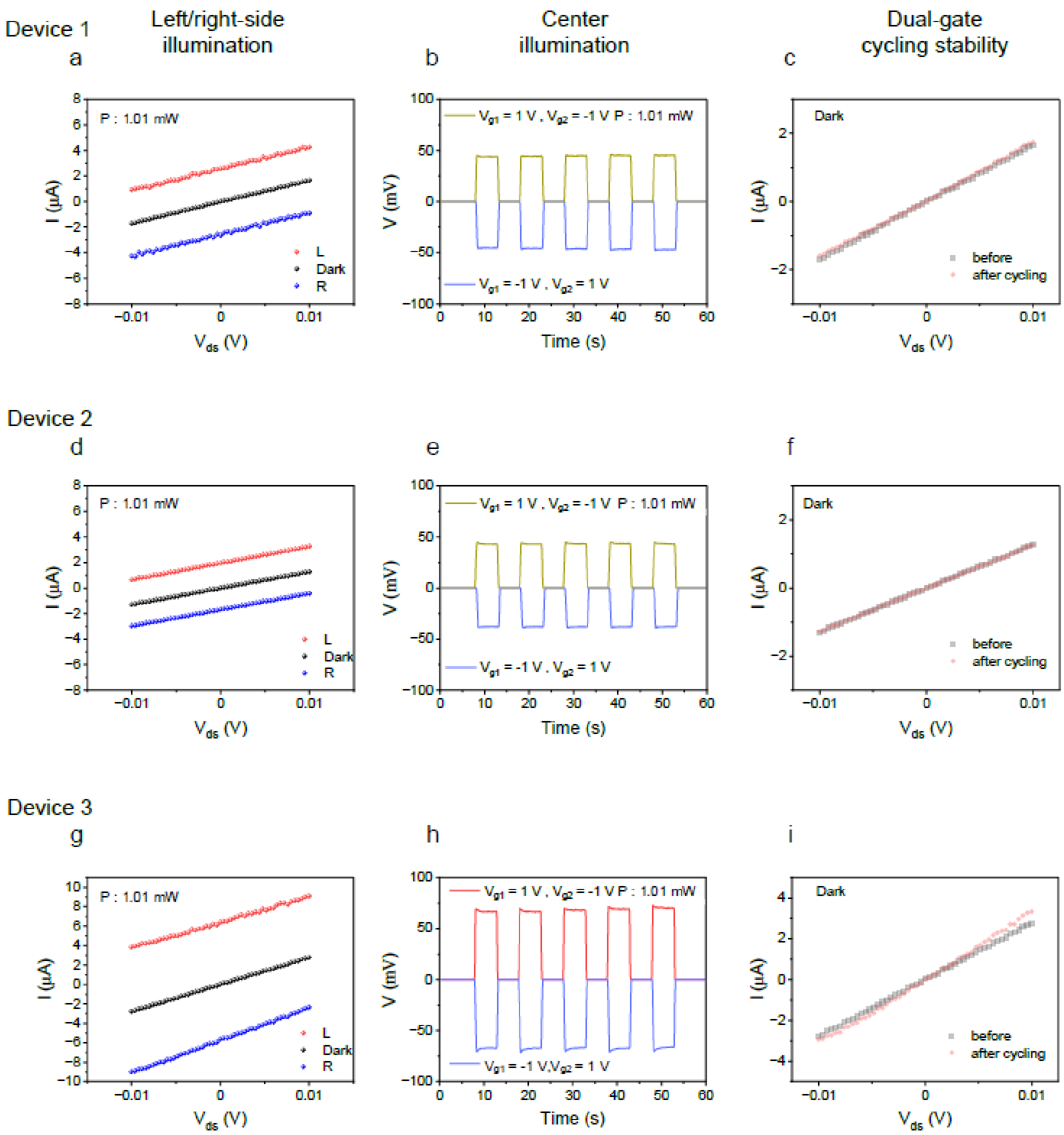

Figure S16. Device-to-device reproducibility of photothermoelectric response and dual-gate cycling stability for Devices 4–6. (a,d,g) Source-drain I-V characteristics of Devices 4-6 measured in the dark and under 4 μm illumination with an incident power of 1.01 mW focused near the left or right contact region. The opposite horizontal shifts under left- and right-side illumination indicate polarity-reversible PTE response. (b,e,h) Centre-illumination photovoltage transients measured at V_{ds} = 0 V under antisymmetric dual-gate programming. The photovoltage polarity reverses when the gate polarity is reversed. (c,f,i) Dark I-V characteristics measured before and after repeated dual-gate cycling, showing stable electrical behaviour under the tested programming conditions. (a,d,g) Source-drain I-V characteristics of Devices 4-6 measured in the dark and under

4 μm illumination with an incident power of 1.01 mW focused near the left or right contact region. The opposite horizontal shifts under left- and right-side illumination indicate polarity-reversible PTE response. (b,e,h) Centre-illumination photovoltage transients measured at V_{ds} = 0 V under antisymmetric dual-gate programming. The photovoltage polarity reverses when the gate polarity is reversed. (c,f,i) Dark I-V characteristics measured before and after repeated dual-gate cycling, showing stable electrical behaviour under the tested programming conditions.

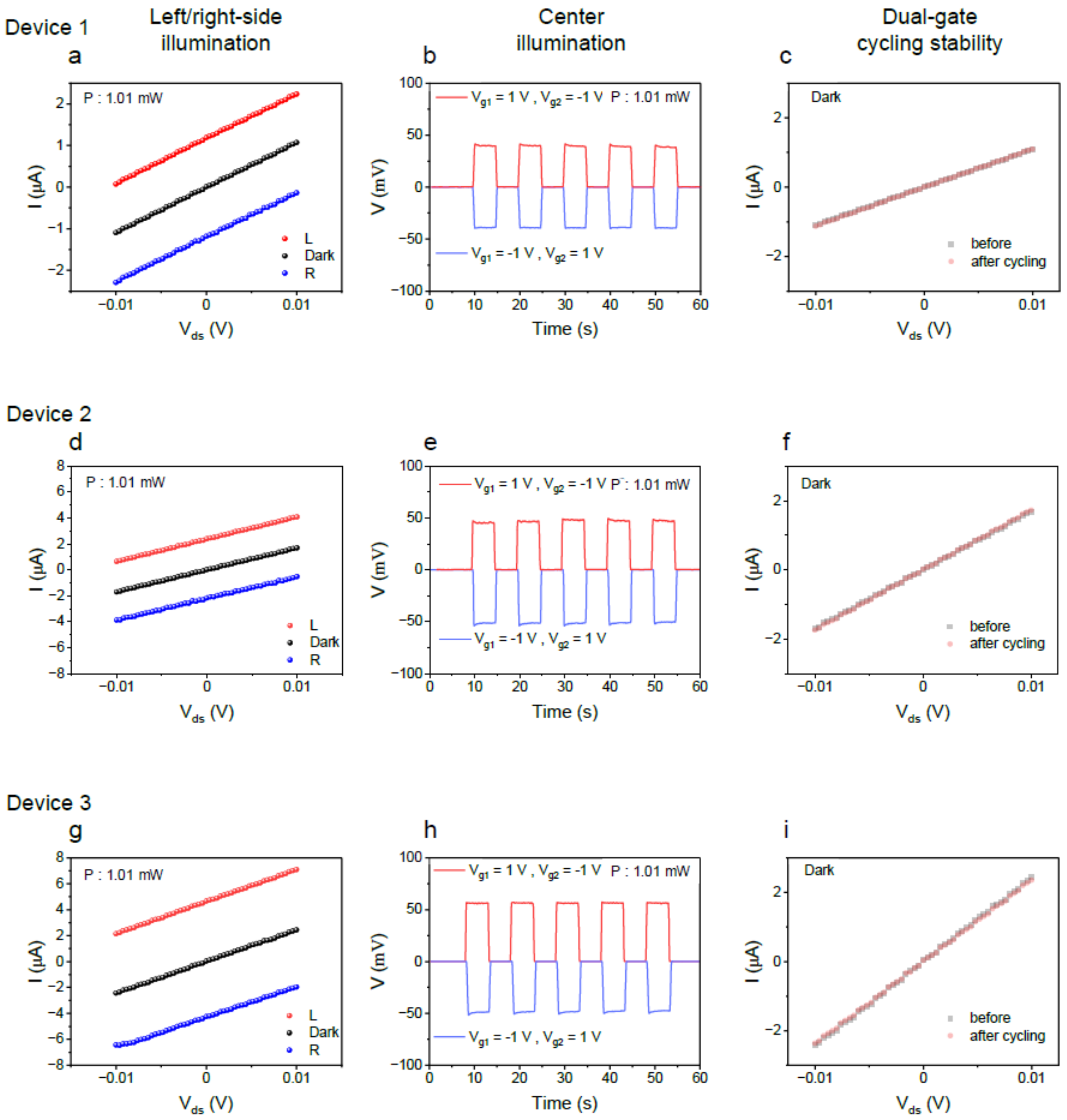

Figure S17. Device-to-device reproducibility of photothermoelectric response and dual-gate cycling stability for Devices 7–9. (a,d,g) Source-drain I-V characteristics of Devices 7-9 measured in the dark and under 4 μm illumination with an incident power of 1.01 mW focused near the left or right contact region. The opposite horizontal shifts under left- and right-side illumination indicate polarity-reversible PTE response. (b,e,h) Centre-illumination photovoltage transients measured at $V_{ds} = 0$ V under antisymmetric dual-gate programming. The photovoltage polarity reverses when the gate polarity is reversed. (c,f,i) Dark I-V characteristics measured before and after repeated dual-gate cycling, confirming the short-term electrical stability of the reinforced contact configuration.

(a,d,g) Source-drain I-V characteristics of Devices 7-9 measured in the dark and under 4 μm illumination with an incident power of 1.01 mW focused near the left or right contact region. The opposite horizontal shifts under left- and right-side illumination indicate polarity-reversible PTE response. (b,e,h) Centre-illumination photovoltage transients measured at V_{ds} = 0 V under antisymmetric dual-gate programming. The photovoltage polarity reverses when the gate polarity is reversed. (c,f,i) Dark I-V characteristics measured before and after repeated dual-gate cycling, confirming the short-term electrical stability of the reinforced contact configuration.

Table S1. Performance comparison of infrared detectors based on different materials and enhancement strategies

	Enhancement strategies	materials	Enhancement mechanism	Response time	Responsivity	Spectral range	NEP (pW Hz$^{1/2}$)	Ref.
Photothermal conversion enhancement	Localized optical field	graphene	Plasmonic antenna	17 ns	27 mA/W	6.1–10 μm	82	1
		graphene	Dipolar antenna	30 ns	14 mA/W	1.8−4.2 THz	80	2
		BP	Plasmons enhancement	3.8 μs	300 V/W, V_d = 3 V	0.15 THz	1 000	3
		Bi_2Te_3/Sb_2Te_3	Plasmons enhancement	0.3 ms	38 V/W (at 646 nm)	500–750 nm	-	4
Thermoelectric process enhancement	Composite materials enhancement	graphene	LSG/$CsPbBr_3$ composite	18 ms	100 mA/W (at 405 nm)	0.4-118 μm	10 000	5
		$SrTiO_3$/CuNi	Semi-suspended structure	0.47 s	4.8 V/W	0.325-10.67 μm	920	6
	Seebeck coefficient manipulation	MoS_2	Bias voltage	-	9.6, 1 V/W	532,700 nm	-	7
		Graphene/SiN	Dual-gate	23 ms	7–9 V/W (at 10.6 μm)	10.6 μm	-	8
		BP	Back gate	-	0.15 V/W	0.298 THz	40 000	9
		graphene	Local electrostatic gate	-	1.5 mA/W	600 nm	-	10
		$SrTiO_3$	Suspended structure	10 μs	3 mA/W, 15 V/W	0.4-10.67 μm	250	**This Work**
	Temperature gradient optimization	$PdSe_2$	Asymmetric contact	50 μs	13 V/W	4.6−10.5 μm	7 000	11
		$SrTiO_3$	Semi-suspended structure	1.52 s	1.2 V/W	0.325-10.67 μm	34 000	12
		$SrTiO_3$/CuNi	Semi-suspended structure	0.47 s	4.8 V/W	0.325-10.67 μm	920	6
		$SrTiO_3$	Suspended structure/dual-gate	10 μs	10 mA/W, 50 V/W	0.4-10.67 μm	25	**This Work**
Conventional materials		HgCdTe (MCT), VIGO PVI-4TE-10.6	TE-cooled photovoltaic detector	25 ns	0.7 A/W at 10.6 μm	3–12 μm	50	13
		Thermopile, Excelitas TPD 1T 0623	Commercial thermopile with IR long-pass filter	27 ms	33 V/W	7.5–13.5 μm	1100	14
		VO_x microbolometer FPA, Teledyne FLIR Boson	Commercial uncooled microbolometer array	8 ms	NETD: ≤ 20–60 mK	8–14 μm	NETD: ≤ 20–60 mK	15

Supplementary Note 1. Mechanistic Interpretation of Thin-Film $SrTiO_3$ for Enhanced Photothermoelectric Response and Programmability

The photothermal response of a thermoelectric detector is governed by both the temperature rise in the absorbing region and the spatial Seebeck-coefficient profile. For a suspended membrane, reducing the $SrTiO_3$ thickness decreases the areal heat capacity, $\rho C_p h$, where ρ is the mass density, C_p is the specific heat capacity and h is the membrane thickness. Under transient illumination, the local temperature rise can be approximated as $\Delta T \approx \eta P_{abs} \Delta t/(\rho C_p A h)$, where η is the optical absorptance, P_{abs} is the absorbed optical power, A is the illuminated area and Δt is the illumination duration. Thus, thinning the $SrTiO_3$ membrane increases the temperature rise under otherwise identical illumination conditions.

The response speed, however, is not determined by thickness alone. The suspended device is better described by an effective thermal circuit, $\tau_{th} = C_{th}/G_{th}$, where C_{th} is the heat capacity of the illuminated suspended membrane region and G_{th} is the total thermal conductance to the surroundings. G_{th} includes heat spreading through the metal electrodes, air-gap conduction, interfacial thermal resistance and radiative or convective losses. Therefore, reducing the membrane thickness is a key factor for faster response, but the absolute response time is set by the complete suspended-device thermal circuit. This interpretation is supported by thickness-dependent measurements, where suspended $SrTiO_3$ membranes with representative thicknesses of 146 nm, 206 nm and 367 nm show transient response times of approximately 6 μs, 16 μs and 30 μs, respectively (Fig. S13).

In addition to thermal improvements, the thin-film architecture enables efficient electrostatic control of the Seebeck profile. The reduced thickness enhances the gate-to-channel capacitance and strengthens electrostatic coupling, which allows local modulation of carrier concentration via gating. This enables spatial control of the Seebeck coefficient, a critical capability for implementing programmable thermoelectric detectors. According to the Mott relation:

$$S = -\frac{\pi^2 k_B^2 T}{3e} \cdot (\frac{dIn\sigma}{dE})_{E_F},$$

where σ is the electrical conductivity and E_F is the Fermi level, electrostatic tuning of the Fermi energy modulates S in a predictable manner. This reconfigurability, combined with the fast and strong photoresponse enabled by thin-film $SrTiO_3$, provides a versatile platform for dynamic infrared detection and in-sensor computing architectures.

Supplementary Note 2. Linear approximation of dual-gate-programmed PTE responsivity

The gate-programmed PTE responsivity originates from the Seebeck contrast between the two gated regions of the suspended $SrTiO_3$ channel. Under center-channel illumination, the voltage responsivity can be expressed phenomenologically as:

$$V_{ph}\left(V_{g1}, V_{g2}\right) = \Delta T(S_L(V_{g1}) - S_R(V_{g2})),$$

where ΔT is the illumination-induced temperature difference and S(Vg) is the effective local Seebeck coefficient under gate bias Vg. The corresponding voltage responsivity is:

$$R_V\left(V_{g1}, V_{g2}\right) = A(S_L(V_{g1}) - S_R(V_{g2})),$$

where P is the incident optical power and A = ΔT/P.

For small gate excursions, S(Vg) can be expanded around zero gate bias:

$$S(V_g) = S(0) + S_1 V_g + S_2 V_g^2 + S_2 V_g^3 + \ldots.$$

Thus,

$$R_V\left(V_{g1}, V_{g2}\right) = K_1(V_{g1} - V_{g2}) + K_2(V_{g1}^2 - V_{g2}^2) + K_3(V_{g1}^3 - V_{g2}^3) + \ldots.$$

Within the measured low-bias range, the leading-order term captures the dominant trend:

$$R_V \approx K\left(V_{g1} - V_{g2}\right).$$

This relation explains the near-zero response along the $V_{g1} = V_{g2}$ diagonal and the sign-reversible response under antisymmetric gating. The measured two-dimensional responsivity map R_V (V_{g1}, V_{g2}) is shown in Fig. S9. The response under $V_{g1} = -V_{g2}$ is plotted in Fig. 3f and fitted using the leading-order empirical relation. Over a wider gate range, weak nonlinearity can be captured by including the cubic correction term:

$$R_V \approx K_1\left(V_{g1} - V_{g2}\right) + K_3(V_{g1}^3 - V_{g2}^3).$$

This empirical treatment avoids assigning the measured response to a specific free-electron-like Seebeck exponent and instead uses the experimentally measured gate dependence to describe the programmable PTE response.

Supplementary Note 3. Noise-limited estimation of resolvable programmable photovoltage states in $SrTiO_3$ PTE detectors

The number of resolvable photoresponse states was estimated from the experimentally measured current dynamic range and the noise floor of the maximum-current state. The minimum and maximum readout currents were

$$I_{\mathrm{min}} = 9.97565 \times 10^{-9}\ \mathrm{A}$$

and

$$I_{\mathrm{max}} = 9.7356 \times 10^{-6}\ \mathrm{A},$$

respectively, giving a current dynamic range of

$$\Delta I = I_{\mathrm{max}} - I_{\mathrm{min}} = 9.72562 \times 10^{-6}\ \mathrm{A}.$$

To avoid overestimating the state resolution, the fluctuation of the maximum-current state was used as the limiting noise floor. After subtracting the dc component from the steady-state current trace,

$$\sigma_I(t) = I(t) - \langle I(t) \rangle,$$

the residual fluctuation was transformed into the frequency domain. The rms noise current was obtained by integrating the single-sided current-noise power spectral density,

$$i_{\mathrm{n,rms}}^{\mathrm{max}} = \left[\int_{f_1}^{f_2} S_{I,\mathrm{max}}(f)\, df \right]^{1/2}.$$

$f_1 = 0.025$ Hz, $f_2 = 4.04$ Hz

The Fourier-domain integration gives

$$i_{\mathrm{n,rms}}^{\mathrm{max}} = 1.46 \times 10^{-9}\ \mathrm{A}.$$

This value was used as the state-discrimination noise term,

$$\delta = i_{\mathrm{n,rms}}^{\mathrm{max}}.$$

Using a 2δ separation criterion, the number of resolvable photoresponse states was estimated as

$$N_{\mathrm{state}} = \left\lfloor \frac{I_{\mathrm{max}} - I_{\mathrm{min}}}{2\delta} \right\rfloor = 3330.$$

Thus, the device theoretically supports at least approximately 3.3×10^3 noise-limited resolvable photoresponse states under the corresponding readout condition.

Supplementary References

1. Castilla S, Vangelidis I, Pusapati V-V et al. Plasmonic antenna coupling to hyperbolic phonon-polaritons for sensitive and fast mid-infrared photodetection with graphene. *Nat Commun* 2020; **11**: 4872.
2. Castilla S, Terrés B, Autore M et al. Fast and sensitive terahertz detection using an antenna-integrated graphene pn junction. *Nano Lett* 2019; **19**: 2765–73.
3. Wang L, Liu C, Chen X et al. Toward sensitive room-temperature broadband detection from infrared to terahertz with antenna-integrated black phosphorus photoconductor. *Adv Funct Mater* 2017; **27**: 1604414.
4. Mauser KW, Kim S, Mitrovic S et al. Resonant thermoelectric nanophotonics. *Nat Nanotechnol* 2017; **12**: 770–5.
5. Li Y, Zhang Y, Chen Z et al. High-performance black phosphorus photodetectors with asymmetric contacts for broadband and polarization-sensitive detection. *Photon Res* 2020; **8**: 1301–8.
6. Guo X, Lu X, Jiang P et al. $SrTiO_3$/CuNi-heterostructure-based thermopile for sensitive human radiation detection and noncontact human–machine interaction. *Adv Mater* 2022; **34**: 2204355.
7. Buscema M, Barkelid M, Zwiller V et al. Large and tunable photothermoelectric effect in single-layer MoS_2. *Nano Lett* 2013; **13**: 358–63.
8. Hsu AL, Herring PK, Gabor NM et al. Graphene-based thermopile for thermal imaging applications. *Nano Lett* 2015; **15**: 7211–6.
9. Viti L, Hu J, Coquillat D et al. Black phosphorus terahertz photodetectors. *Adv Mater* 2015; **27**: 5567–72.
10. Lemme MC, Koppens FHL, Falk AL et al. Gate-activated photoresponse in a graphene p-n junction. *Nano Lett* 2011; **11**: 4134–7.
11. Dai M, Wang C, Ye M et al. High-performance, polarization-sensitive, long-wave infrared photodetection via photothermoelectric effect with asymmetric van der Waals contacts. *ACS Nano* 2022; **16**: 295–305.

12. Lu X, Jiang P, Bao X. Phonon-enhanced photothermoelectric effect in $SrTiO_3$ ultra-broadband photodetector. *Nat Commun* 2019; **10**: 138.
13. VIGO Photonics. PVI-10.6 detector series datasheet. https://vigophotonics.com/product/hgcdte-photovoltaic-ir-detectors-pvi-4te-10-6-1x1-to8-to66-wznsear-36/ (26 July 2026, date last accessed).
14. Excelitas Technologies. TPiD 1T 0624 thermopile detector datasheet. https://www.excelitas.com/assets/product/document/tpid-1t-0624-datasheet.pdf?file=24668.pdf (26 July 2026, date last accessed).
15. Teledyne FLIR. Boson uncooled LWIR OEM thermal camera module datasheet. https://oem.flir.com/products/boson/ (26 July 2026, date last accessed).
